# High-throughput sequencing of small RNAs and analysis of differentially expressed microRNAs associated with pistil development in Japanese apricot

**DOI:** 10.1186/1471-2164-13-371

**Published:** 2012-08-03

**Authors:** Zhihong Gao, Ting Shi, Xiaoyan Luo, Zhen Zhang, Weibing Zhuang, Liangju Wang

**Affiliations:** 1 College of Horticulture, Nanjing Agricultural University, No 1 Weigang, Nanjing, 210095, P. R. China

**Keywords:** Japanese apricot, microRNA, Pistil abortion, qRT-PCR, Solexa sequencing

## Abstract

**Background:**

MicroRNAs (miRNAs) are a class of endogenous, small, non-coding RNAs that regulate gene expression by mediating gene silencing at transcriptional and post-transcriptional levels in high plants. However, the diversity of miRNAs and their roles in floral development in Japanese apricot (*Prunus mume* Sieb. et Zucc) remains largely unexplored. Imperfect flowers with pistil abortion seriously decrease production yields. To understand the role of miRNAs in pistil development, pistil development-related miRNAs were identified by Solexa sequencing in Japanese apricot.

**Results:**

Solexa sequencing was used to identify and quantitatively profile small RNAs from perfect and imperfect flower buds of Japanese apricot. A total of 22,561,972 and 24,952,690 reads were sequenced from two small RNA libraries constructed from perfect and imperfect flower buds, respectively. Sixty-one known miRNAs, belonging to 24 families, were identified. Comparative profiling revealed that seven known miRNAs exhibited significant differential expression between perfect and imperfect flower buds. A total of 61 potentially novel miRNAs/new members of known miRNA families were also identified by the presence of mature miRNAs and corresponding miRNA*s in the sRNA libraries. Comparative analysis showed that six potentially novel miRNAs were differentially expressed between perfect and imperfect flower buds. Target predictions of the 13 differentially expressed miRNAs resulted in 212 target genes. Gene ontology (GO) annotation revealed that high-ranking miRNA target genes are those implicated in the developmental process, the regulation of transcription and response to stress.

**Conclusions:**

This study represents the first comparative identification of miRNAomes between perfect and imperfect Japanese apricot flowers. Seven known miRNAs and six potentially novel miRNAs associated with pistil development were identified, using high-throughput sequencing of small RNAs. The findings, both computationally and experimentally, provide valuable information for further functional characterisation of miRNAs associated with pistil development in plants.

## Background

Japanese apricot (*Prunus mume* Sieb. et Zucc) is an important economic fruit crop in China and Japan, with more than 200 cultivars in China [1]. Japanese apricot fruit has consistently been one of the most valuable processing materials used in the food and wine-making industries and is believed to contain many physiochemicals beneficial to human health. However, the phenomenon of imperfect flowers is common and seriously affects production yields. The percentage of imperfect flowers depends on the cultivar; the highest is 75.15% and the average is 35% [2]. Imperfect flowers are characterised by pistils below the stamens, withered pistils or the absence of pistils, and hence, they fail to bear fruit [3]. Several environmental factors and physiological processes have been shown to affect pistil development [1,4]. Previous research indicated that several miRNAs and multiple target genes are involved in flower development in model plants [5-12]. More recently, real-time quantitative reverse transcription polymerase chain reaction and *in situ* hybridisation showed that the *PmAG* mRNA was highly-expressed in the sepals, carpels and stamens, and a weak signal was detected in the seeds and nutlets. No expressions were detected in the leaves or petals, but no significant differential was expressed between perfect and imperfect flowers [3]. Meanwhile, comparative proteomic analyses were performed on perfect and imperfect flowers, and several differently-expressed proteins were identified [13]. However, the type of molecular mechanism involved in pistil abortion remains unknown in Japanese apricot.

Small RNAs (sRNAs) are low molecular weight RNAs with regulatory functions. Based on differences in biogenesis and action, sRNAs are grouped into two categories: short interfering RNAs (siRNAs) and microRNAs (miRNAs) [14,15]. MicroRNAs are non-coding RNAs, 21–24 nucleotides (nt) long, which regulate gene expression at the post-transcriptional level [16-18]. In plants, miRNAs are processed from the stem-loop regions of long primary transcripts by a Dicer-like enzyme and are loaded into silencing complexes, where they generally direct the cleavage of complementary mRNAs [17]. Identified in plants less than 10 years ago [19,20], miRNAs are already known to play numerous crucial roles at each major stage of development, typically at the cores of gene regulatory networks, targeting genes that are themselves regulators, such as those that encode transcription factors, suggesting that plant miRNAs are master regulators [18,21]. Among non-transcription factor targets, many miRNAs encode F-box proteins or ubiquitin-conjugating enzymes implicated in targeting selected proteins for proteasomal degradation, indicating miRNAs play a role in regulating protein stability and plant development [22,23]. miR156, miR163, miR169, miR172, miR398 and miR399 play important roles in flowering-time regulation and belong to ambient temperature-responsive miRNAs in plants [9,24]. miR172 has also acquired specialised species-specific functions in other aspects of plant development, such as cleistogamy and tuberisation [6].

The fact that a large number of the known miRNAs in the plant kingdom, from mosses and ferns to higher flowering plants, are evolutionarily conserved has been used as a practical indicator for the identification or prediction of miRNAs using homology searches in other species [25,26]. Recently developed, next-generation, high-throughput sequencing technologies provide a powerful tool for identifying, as well as quantifying, miRNAs. These technologies open up the possibility of exploring sRNA populations in economically important species such as *Arabidopsis thaliana*[27,28], *Oryza sativa*[29,30], *Populus trichocarpa*[31,32], *Vitis vinifera*[33], *Arachis hypogaea*[34], *Citrus sinensis*[35], *Citrus trifoliate*[36], *Medicago truncatula*[37,38], *Glycine max*[39], *Carthamus tinctoriusCucumis sativus*[40], *Rehmannia glutinosa*[41] and others. By means of high-throughput sequencing, miR164 and miR169 were shown to be drought-responsive miRNAs in *Medicago truncatula*[37]. miR167, miR1857 and miR172a are involved in the mutant trait formation of lycopene accumulation in sweet orange [35].

Although miRNAs have been extensively studied in the past, there has be no systematic examination of miRNAs performed on the Japanese apricot. To investigate the role of miRNAs on the pistil development of Japanese apricot, high-throughput sequencing technology (Solexa) was employed to survey sRNA populations from perfect and imperfect flower buds.

## Results

### High-throughput sequencing of small RNAs from Japanese apricot flower bud tissue

**Figure 1:**
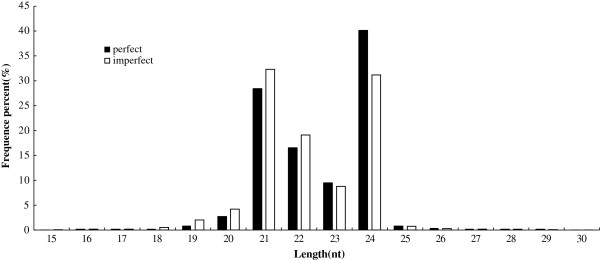
Length distribution of the small RNA library in Japanese apricot perfect and imperfect libraries.

**Figure 2:**
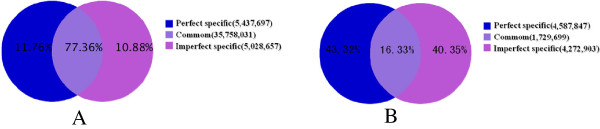
**Summary of common and specific sequences between perfect and imperfect libraries.** (**A**)Total sRNAs and (**B**)Unique sRNAs.

To identify miRNAs involved in the development of Japanese apricot flowers, Solexa sequencing was used on libraries of small RNAs from perfect and imperfect flower buds. A total of 22,561,972 and 24,952,690 reads were obtained from perfect and imperfect flower bud libraries, respectively, after filtering out those reads without small RNA sequences, ranging from 18 to 30 nt in length (Figure 1), of which the majority were 19–25 nt long. The most abundant small RNAs in the perfect and imperfect libraries were 21 nt and 24 nt long, respectively. The distribution of 21 nt small RNAs was approximately 28.35% and 32.31% in perfect and imperfect libraries, respectively, while the distribution of 24 nt small RNAs was approximately 40.08% and 31.17% in the perfect and imperfect libraries, respectively. After removal of the adaptor, insert, poly(A) and short RNAs of <18 nt, 21,985,053 and 24,239,332 redundant clean reads were obtained from the perfect and imperfect flower buds, respectively, including rRNA, tRNA, snRNA, snoRNA, miRNA and several other unannotated reads (Tables 1 and 2). Of these clean reads, 11.76% (unique, 43.32%) were perfect-specific, 10.88% (unique, 40.35%) were imperfect-specific, and 77.36% (unique, 16.33%) were present in both (Figure 2). The average number of common sequence frequency was 20.67, while that of library-specific reads was not more than 1.2 (Table 3). Among the sRNAs sequenced, unannotated redundant reads accounted for 77.83% and 74.25% for perfect and imperfect flower buds, respectively.

**Table 1:** Summary of data cleaning produced by small RNA sequencing (perfect and imperfect flower buds)

**Type**	**Perfect**		**Imperfect**	
	**Count**	**Percent (%)**	**Count**	**Percent (%)**
Total reads	22,561,972		24,952,690	
High quality	22,135,703	100%	24,466,679	100%
3′ adapter null	53,592	0.24%	49,464	0.20%
Insert null	6,598	0.03%	8,281	0.03%
5′ adapter contaminants	36,203	0.16%	58,925	0.24%
Smaller than 18nt	48,147	0.22%	107,094	0.44%
Poly(A)	6,110	0.03%	3,583	0.01%

**Table 2:** Distribution of small RNAs among different categories (perfect and imperfect flower buds)

**Category**	**Perfec0074**		**Imperfect**	
	**Unique reads**	**Redundant reads**	**Unique reads**	**Redundant reads**
Total	6,317,546 (100%)	21,985,053 (100%)	6,002,602 (100%)	24,239,332 (100%)
rRNA	55,021 (0.87%)	1,990,779 (9.06%)	63,243 (1.05%)	2,844,574 (11.74%)
snRNA	4,443 (0.07%)	31,490 (0.14%)	5,145 (0.09%)	40,170 (0.17%)
snoRNA	1,376 (0.02%)	6,672 (0.03%)	1,445 (0.02%)	7,517 (0.03%)
tRNA	10,502 (0.17%)	364,461 (1.66%)	11,885 (0.20%)	698,413 (2.88%)
miRNA	26,734 (0.42%)	2,479,659 (11.28%)	30,931 (0.52%)	2,649,934 (10.93%)
Unannotated	6,219,470 (98.45%)	17,111,992 (77.83%)	5,889,953 (98.12%)	17,998,724 (74.25%)

**Table 3:** Small RNA sequences and mean frequencies in both perfect and imperfect libraries

**Class**	**Unique sRNAs**	**Total sRNAs**	**Mean frequency**
Total sRNAs	10590449	46224385	4.36
Perfect and imperfect common	1729699	35758031	20.67
Perfect specific	4587847	5437697	1.19
Imperfect specific	4272903	5028657	1.18

### Identification of known miRNAs and evolutionary conservation

To identify the known miRNAs, the sRNA library was BLASTN searched for known mature plant miRNAs in miRBase 17.0 (April 2011). Following the BLASTN searches and further sequence analysis, 61 unique sequences, belonging to 24 families, in the perfect and imperfect libraries were found to be orthologues of known miRNAs from other plant species, which had previously been deposited in the miRBase database (Table 4).

**Table 4:** Known miRNAs in perfect and imperfect libraries

**Family**	**miRNA name**	**Perfect**		**Imperfect**	
		**Counts**	**Normalised**	**Counts**	**Normalised**
MIR156	miR156a	45352	2062.86	61361	2531.46
	miR156b	26572	1208.64	29886	1232.95
	miR156c	145205	6604.71	210543	8686.01
	miR156f	118	5.37	188	7.76
	miR156h	0	0.01	4	0.17
	miR156k	25	1.14	46	1.90
	miR157a	735314	33446.09	658343	27160.11
	miR157d	8866	403.27	8738	360.49
MIR159	miR159a	18682	849.76	38726	1597.65
	miR319	21	0.96	171	7.05
	miR319a	121	5.50	1172	48.35
	miR319c	6	0.27	7	0.29
	miR319e	1	0.05	40	1.65
	miR319g	0	0.01	5	0.21
MIR160	miR160a	110	5.00	808	33.33
MIR162	miR162	1	0.05	4	0.17
	miR162a	718	32.66	617	25.45
MIR164	miR164a	6256	284.56	4134	170.55
	miR164f	63	2.87	52	2.15
MIR166	miR166	60	2.73	85	3.51
	miR166a	222689	10129.11	281639	11619.09
	miR166h	1369	62.27	2013	83.05
MIR167	miR167-3p	810	36.84	691	28.51
	miR167a	151440	6888.32	127360	5254.27
	miR167d	52161	2372.57	45113	1861.15
	miR167f	61643	2803.86	44482	1835.12
MIR168	miR168a	64099	2915.57	82261	3393.70
MIR169	miR169b	510	23.20	538	22.20
	miR169e	10	0.45	6	0.25
	miR169g	11	0.50	1	0.04
	miR169h	18	0.82	10	0.41
MIR171	miR171	483	21.97	630	25.99
	miR171b	1578	71.78	1994	82.26
	miR171d	1	0.05	0	0.01
	miR171f	13	0.59	15	0.62
	miR171b-3p	2195	99.84	2831	116.79
	miR171l	4	0.18	9	0.37
MIR172	miR172a	13173	599.18	10393	428.77
	miR172b	5	0.23	0	0.01
	miR172e	181	8.23	181	7.47
	miR172g	123	5.59	102	4.21
MIR2111	miR2111a	0	0.01	5	0.21
MIR390	miR390a	18872	858.40	32445	1338.53
	miR390a-3p	602	27.38	1207	49.80
MIR393	miR393	221	10.05	603	24.88
	miR393a	2	0.09	77	3.18
	miR393b	4	0.18	3	0.12
MIR394	miR394a	24	1.09	301	12.42
MIR395	miR395a	14	0.64	94	3.88
	miR395b	1	0.05	2	0.08
MIR396	miR396a	2046	93.06	3149	129.91
	miR396b	869	39.53	1194	49.26
MIR398	miR398	0	0.01	1	0.04
	miR398a	13	0.59	16	0.66
	miR398b	7	0.32	12	0.50
MIR399	miR399a	0	0.01	7	0.29
	miR399f	29	1.32	45	1.86
MIR403	miR403	325	14.78	510	21.04
MIR408	miR408	19	0.86	14	0.58
MIR535	miR535a	9743	443.16	12158	501.58
MIR827	miR827a	22512	1023.97	21786	898.79
MIR828	miR828a	48	2.18	48	1.98

**Figure 3:**
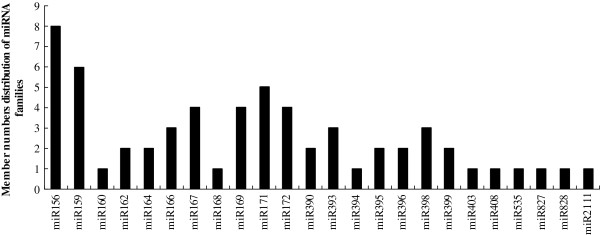
Member numbers of identified miRNAs in each conserved miRNA family in Japanese apricot.

The number of members in differently-conserved miRNA families was analysed (Figure 3). A majority of the 24 miRNA families contained several members, and three families (miR156, miR159 and miR171) possessed multiple members, with 8, 6 and 5 members, respectively. Nine miRNA families, namely miR160, miR168, miR394, miR403, miR408, miR535, miR827, miR828 and mir2111, had only one member.

**Figure 4:**
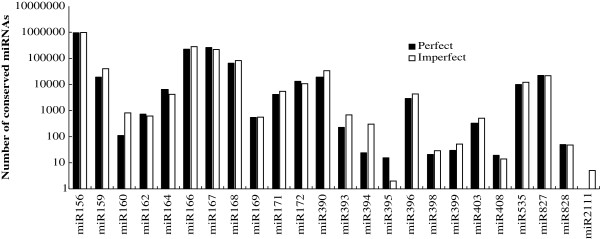
Counts of identified miRNAs in each conserved miRNA family in Japanese apricot.

The counts of reads for known miRNA families were also analysed, and it was found they indicated large divergences in expression frequency among these miRNAs. The number of reads for conserved miRNAs sequenced from the same, or different, miRNA families also varied drastically, with miR156, miR166 and miR167 represented most frequently in the two libraries. Of these, miR156 was represented the most frequently, with 961,452 and 969,109 copies in the perfect and imperfect libraries, respectively. miR156, miR169, miR827 and miR828 were moderately abundant in the two libraries, while miR2111 was absent from the perfect library. It appeared, therefore, that the miRNA population present in the perfect library differed from that present in the imperfect library to some extent (Figure 4).

**Figure 5:**
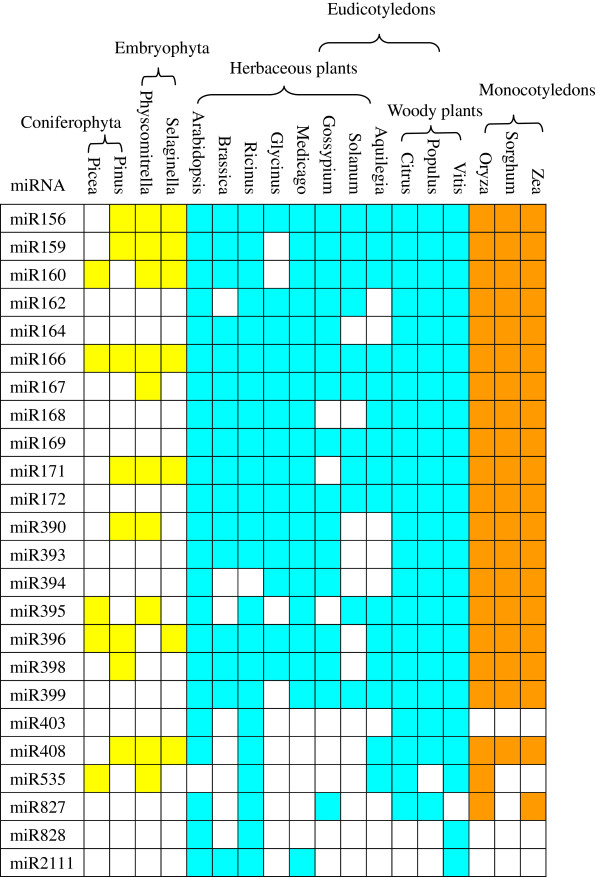
**Known miRNAs from Japanese apricot, designated as** ***pmu*** **on the first column, and their homologs in other plant species.**

To investigate the evolutionary roles of these known miRNAs, extensive comparisons were performed against known miRNAs in other plant species, including *Physcomitrella, Selaginella, Picea, Pinus, Arabidopsis, Brassica, Ricinus, Glycine, Medicago, Gossypium, Solanum, Aquilegia, Citrus, Populus, Vitis, Oryza, Sorghum* and *Zea* (Figure 5). Among the miRNA sequences obtained from Japanese apricot, members in five of families (miR403, miR535, miR827, miR828 and miR2111) showed a lack of conservation of sequence identity compared to orthologues from 18 other plant species. Generally, Japanese apricot miRNAs had corresponding homologues in at least two plant species. Japanese apricot, *Arabidopsis* and *Vitis vinifera* shared 23 conserved miRNA families. Twelve (miR156, miR159, miR160, miR166, miR167, miR171, miR390, miR395, miR396, miR398, miR408 and miR535) out of 24 families had orthologues in Coniferophyta and Embryophyta, indicating that these 12 Japanese apricot miRNA families are ancient. Twelve (miR162, miR164, miR168, miR169, miR172, miR393, miR394, miR399, miR403, miR827, miR828 and miR2111) out of 24 families only had homologues in angiosperm, indicating that these 12 Japanese apricot miRNA families are recent. In addition, miR403, miR828 and miR2111 only had homologues in eudicotyledons, suggesting that these miRNAs are probably involved in the regulation of specific development, such as the development of embryos and cotyledons.

### Identification of potentially novel miRNAs/new members of known miRNA families and nucleotide bias

**Figure 6:**
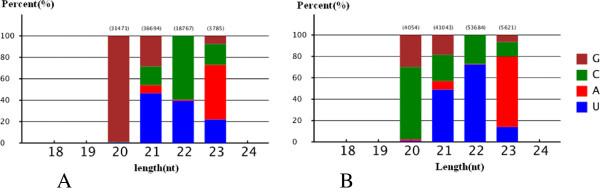
**Novel miRNA candidates first nucleotide bias in Japanese apricot.** (**A**) Perfect library and (**B**) Imperfect library.

The Mireap prediction software developed by the Beijing Genomics Institute (Shenzhen, China) was used to identify potentially novel miRNAs and new members of known miRNA families by exploring the secondary structure based upon a trans-species alignment of apricot small RNA data against the peach (*Prunus persica*) genome. After removal of the conserved miRNAs, sequences were aligned with the peach genome sequence. A total of 405 and 423 miRNA candidates were obtained from the perfect and imperfect flower bud libraries, respectively. The prediction of potentially novel miRNA/new members of known miRNA families candidates was summarised, including the base bias on the first position among small RNA candidates of certain lengths. Based on this summary, prediction accuracy could be assessed according to the base bias of known miRNAs. The majority of these novel miRNA candidates had lengths of 21 and 22 nt, and started with a 5′ U (Figure 6A and B).

Among the miRNA candidates, 61 were identified with complementary miRNA*s (fold-back structures in Additional files 1 and 2), indicating that these candidate miRNAs were most likely new in Japanese apricot, and several potentially novel ones may be unique to Japanese apricot or *Prunus* plants. The counts of several miRNA*s of these potentially novel miRNAs were low (Table 5). Nevertheless, no requirement for counts of miRNA*s was given in the most recent criteria for the annotation of plant miRNAs [42]. Therefore, these miRNAs were still considered to be potentially new. Candidate miRNAs with only one miRNA* have been identified as new miRNAs in rice and leguminous plants. The minimum free energy (MFE) for the hairpin structures of miRNA precursors was lower than −18 kcal/mol, and the length of new miRNA precursors ranged from 74 nt to 377 nt (Table 5).

**Table 5:** Novel miRNAs/new members of known miRNA families in perfect and imperfect libraries

**miRNA**	**Length of mature(nt)**	**Sequence(5′–3′)**	Counts of miRNAs/miRNA*s	**Location**	**Length of precursors(nt)**	**Arm**	**MFE (kcal/mol)**
miR6257	21	TCTTAACTGTTGGATTAGGCT	22/1	scaffold_10:708566:708677:−	112	5′	−26.7
miR6258	21	TTCCAGCTGTAAAGATCAAGA	5/5	scaffold_1:230373:230544:+	172	5′	−88.7
miR6259	21	TAGAAAAATACGGGCGATAAA	6/1	scaffold_1:37406755:37406880:+	126	3′	−53.6
miR6260	21	TGGAGTGAGAGAATGGGAGGT	14/2	scaffold_1:40569695:40569796:+	102	3′	−50.4
miR6261	22	AAGTGATTATATGGAGAAGCAC	13/1	scaffold_1:46392103:46392176:+	74	3′	−26.5
miR6262	21	TCTTTAGAAAGTTAGAATTGT	5/1	scaffold_1:23904565:23904750:−	186	5′	−67
miR6263	21	AAGTGGACAAAAGGGGAGTGG	16/2	scaffold_2:16774627:16774733:+	107	3′	−24.9
miR6264	21	ATGCCTATGGACACGTGTCAA	6/1	scaffold_2:10473541:10473644:−	104	3′	−56.02
miR6265	23	TTGAACTTTGACCCGATTCGCAT	15/1	scaffold_3:13798983:13799084:+	102	5′	−36.77
miR6266a	21	TAAATGCAGGGGCAAAATGAT	149/4	scaffold_4:6953607:6953883:+	277	5′	−69.81
miR6266b	21	TAAATGCAGGGGCAAAATGAT	109/2	scaffold_4:8221370:8221603:+	234	5′	−60.63
miR6266c	21	TAAATGCAGGGGCAAAATGAT	106/1	scaffold_7:12665062:12665327:−	266	5′	−59.43
miR6267a	21	TAGAGAGGTGGTACAATTGTG	45/1	scaffold_4:9720663:9720766:+	104	3′	−69.6
miR6267b	21	ATTAGAGAGGCGGTAAACAAT	5/1	scaffold_4:13686619:13686731:−	113	3′	−35.9
miR6268	21	TGAGGAGATGGAGAGTAGATA	244/3	scaffold_4:16645739:16645874:+	136	5′	−77.7
miR6269	21	TGTGAATAGTGATTGCCATGG	18/5	scaffold_4:20136895:20136989:+	95	5′	−36.7
miR6270	21	TTCTGGTATTGGAATTTCATT	259/26	scaffold_4:30105651:30106027:+	377	3′	−117.7
miR6271	21	TCAAGATTGAGAGATATAATG	24/3	scaffold_4:8916468:8916590:−	123	3′	−34.3
miR6272	21	TAGCTGTAAATGAGTGTTTTT	5/1	scaffold_4:19289821:19289925:−	105	5′	−25.8
miR6273	21	AATGCAGCATGATTTTTTTTT	81/63	scaffold_5:3421010:3421132:+	123	3′	−42.2
miR6274	23	TATTTTGCTATCTTCGGGCAATA	15/2	scaffold_5:3414706:3414834:−	129	5′	−35.8
miR6275	22	AGTGGAAGTAGCAAGGGGAAGC	14/1	scaffold_5:10005396:10005502:−	107	3′	−67.6
miR6276	21	AAAGGCTCATACAAATATTCC	14/3	scaffold_6:8093383:8093491:−	109	5′	−21.7
miR6277	21	TGTGTGTGGAAAGAGCGAGAC	1661/5	scaffold_6:28212589:28212684:−	96	3′	−47.16
miR6278	22	TGAACCTTGTGTACAAATTGGC	16/3	scaffold_7:7789275:7789372:+	98	3′	−41.9
miR6279	21	TAGACAAGAATTCCAGAGACC	9/1	scaffold_7:16179849:16180001:+	153	3′	−31.4
miR6280	21	TTGGCAGTAAGATTTTTGGTG	15/2	scaffold_7:8897336:8897500:−	165	5′	−42.1
miR6281	21	GTTAGAGATAGAGAGAGTGAG	60/21	scaffold_8:277682:277841:+	160	5′	−86
miR6282	23	GTTGATCGATGTGGGATGTTACA	8/1	scaffold_8:11190431:11190772:+	342	5′	−93.3
miR6283	21	CAAAAGGGGAGTGGGAAAATC	33/2	scaffold_8:19627180:19627307:+	128	3′	−64.9
miR6284	21	TTTGGACCATGGATGAAGATT	5128/2	scaffold_8:20948100:20948196:+	97	3′	−31.9
miR6285	22	TAGTGAAGTTTGAATTAGGGCT	2195/3	scaffold_8:16684630:16684710:−	81	5′	−38
miR6286	23	TTTGAACCATTGGATCGTAGTTA	8/1	scaffold_14:309411:309508:−	98	5′	−21.8
miR6287	21	CAAGAAGTGGAAGTTTTGGGC	5/1	scaffold_1:17236128:17236204:−	77	5′	−31.3
miR6288	21	GAAAATGACAAGTGGCTAGTT	27/13	scaffold_2:18743450:18743549:+	100	3′	−51.3
miR6289	21	TCCTTTGAATGGTTAGGCTCA	7/1	scaffold_3:4077260:4077364:−	105	3′	−51.2
miR6290	23	TGAATGAGTTCAGAGATCGTGTA	17/12	scaffold_4:28016862:28016957:+	96	3′	−18.7
miR6291a	21	CTTACCACATTTTTATACCAT	67/1	scaffold_4:8758152:8758281:−	130	5′	−66.31
miR6291b	21	CTTACCACATTTTTATACCAT	67/4	scaffold_7:5853383:5853533:+	151	5′	−41.6
miR6292	21	TATCTTTTAATCGTTAGATCA	18/1	scaffold_4:11582347:11582421:−	75	5′	−29.99
miR6293	21	TAAGAGGCTGATGACTAAAAC	355/9	scaffold_5:2922263:2922370:+	107	5′	−59.8
miR6294	21	TGGTGTAGGCTAATCACAATC	7/1	scaffold_5:18324006:18324209:+	204	3′	−89.9
miR6295	21	GAGGACAGAAGATGATTCAGC	42/10	scaffold_6:25684504:25684833:+	330	3′	−155.3
miR6296	21	TAAGGCCCTTAGATGAGACCC	10/1	scaffold_6:28262446:28262576:+	131	3′	−56
miR6297a	23	AATAATTTTTCGTCGCGCAAAAT	10/1	scaffold_8:6395625:6395769:+	145	3′	−63.2
miR6297b	23	GATGTATTGTCGTCGCGCAAAGT	11/11	scaffold_8:2802801:2802903:−	103	3′	−54.6
miR171a	21	TGATTGAGCCGTGCCAATATC	1651/29	scaffold_3:16557250:16557364:+	115	3′	−58.4
miR171c	21	TGATTGAGCCGTGCCAATATC	1586/1	scaffold_3:21505898:21505996:−	99	3′	−46.3
miR171e	21	TTATTGAACCGGACCAATATC	10/1	scaffold_3:16557258:16557354:−	97	3′	−39.8
miR171g	21	TGATTGAGCCGTGCCAATATC	3634/3	scaffold_5:13607844:13607938:−	95	3′	−46.5
miR171h	21	TTGAGCCGCGTCAATATCTCC	1141/43	scaffold_3:16525596:16525722:+	127	3′	−44.32
miR319b	22	TAGCTGCCGAGTCATTCATCCA	71/30	scaffold_5:11602974:11603095:−	122	5′	−49.26
miR394	21	AAGCGTTTCTTACAGAGTTTA	130/4	scaffold_1:32136105:32136248:−	144	5′	−65
miR477a	21	GTTGGGGGCTCTTTTGGGACG	161/28	scaffold_3:9747726:9747847:−	122	3′	−58.5
miR477b	21	GTTGGGGGCTCTTTTGGGACG	161/28	scaffold_3:9752784:9752905:−	122	3′	−62.1
miR482a	22	TTTCCGAAACCTCCCATTCCAA	1293/75	scaffold_1:29646062:29646181:+	120	3′	−47.2
miR482b	22	CTTCCCAAACCTCCCATTCCTA	4677/518	scaffold_1:29648520:29648655:+	136	3′	−55.5
miR482c	20	GGAATGGGCTGTTTGGGATG	30342/30319	scaffold_3:10579287:10579407:+	121	5′	−63.6
miR482d	22	CCTCCCATGCCACGCATTTCTA	20734/3022	scaffold_8:10608388:10608531:−	144	3′	−59.3
miR482e	22	TTGCCTATTCCTCCCATGCCAA	210/2	scaffold_1:29646743:29646870:+	128	3′	−55
miR828	21	TCATTTCAGCAAGCAGCGTTA	1491/1	scaffold_2:25971195:25971319:−	125	3′	−55.9

### Differentially expressed miRNAs between perfect and imperfect flower bud libraries

Small RNAs in the perfect and imperfect libraries were enriched for lengths of 21–24 nt (Figure 1), a typical range for plant miRNAs. For small RNAs shorter than 23 nt, the imperfect flower buds had higher expression levels than the perfect flower buds, while for small RNAs longer than 23 nt, the opposite was the case. In addition to the different length distributions of small RNAs, the proportions of each type of small RNA in the two libraries were also different (Table 2). The proportion of miRNAs in perfect flower buds was higher than that in imperfect flower buds. However, rRNAs, snRNAs and tRNAs in imperfect flower buds were higher than in perfect flower buds. In summary, the small RNA transcriptomes of the two libraries exhibited certain differences with respect to length distribution and composition.

In order to identify miRNAs related to pistil development, the normalised expressions of miRNAs in the perfect and imperfect small RNA libraries were compared. The miRNAs with changes in expression levels greater than 1.5-fold and *p*-values less than 0.05 are presented in Table 6. The results from high-throughput sequencing showed that seven known miRNAs and six potentially novel miRNAs/new members of known miRNA families were relevant to pistil development (Table 6). Of these 13 sequences, nine demonstrated a greater than three-fold change in expression levels between the perfect and imperfect libraries. Among the six differentially expressed potentially novel miRNAs/new members of known miRNA families, miR6274 and miR482c were perfect specific, while miR6295, mi171d and miR319b were imperfect specific. Three miRNAs were down-regulated, and ten miRNAs were up-regulated in pistil abortion (Table 6).

**Table 6:** miRNAs expressed differentially in perfect and imperfect libraries

**Family**	**miRNA name**	**Perfect**		**Imperfect**		**Fold-change log2(s2/s1)**	**P-value**	**Mode**	**Sig-lable**
		**Counts**	**Normalised**	**Counts**	**Normalised**				
MIR159	miR319	21	0.96	171	7.05	2.88	1.45e − 06	Up	**
	miR319a	121	5.50	1172	48.35	3.14	0	Up	**
	miR319e	1	0.05	40	1.65	5.18	0	Up	**
MIR160	miR160a	110	5.00	808	33.33	2.74	0	Up	**
MIR393	miR393a	2	0.09	77	3.18	5.13	6.41e − 12	Up	**
MIR394	miR394a	24	1.09	301	12.42	3.51	9.05e − 51	Up	**
MIR395	miR395a	14	0.64	94	3.88	2.61	6.82e − 25	Up	**
unknown	miR6274	60	2.73	0	0.01	−8.09	0	Down	**
unknown	miR6268	178	8.10	66	2.72	−1.57	0	Down	**
unknown	miR6295	0	0.01	42	1.73	7.44	1.97e − 37	Up	**
MIR159	miR319b	0	0.01	71	2.93	8.19	4.14e − 99	Up	**
MIR171	miR171d	0	0.01	2097	86.51	13.08	0	Up	**
MIR482	miR482c	30342	1380.12	0	0.01	−17.07	0	Down	**

**Figure 7:**
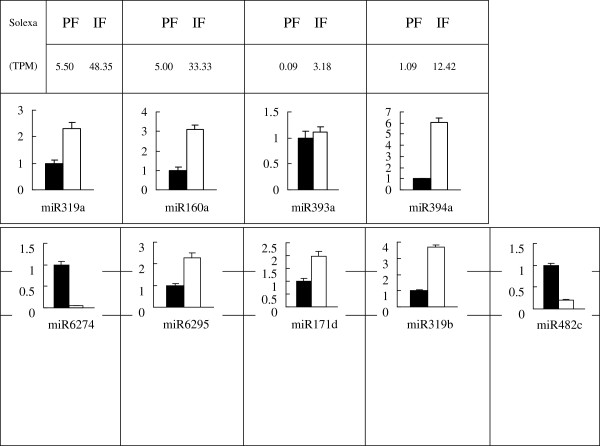
**Expression confirmation of miRNAs in Japanese apricot derived from high throughput sequencing.** Differentially expressed miRNAs expression detected by poly(A) qRT-PCR.

To confirm the differential expression of the miRNAs, the expressions of four of the seven known miRNA genes and five of the six novel miRNA genes were analysed using poly(A) qRT-PCR. The expression level of each gene in the perfect and imperfect flower buds was compared with its abundance in the sequencing data of the sRNA library. The results indicated that nine miRNAs had the same expression patterns in the perfect and imperfect flower buds as those in the sequencing data (Figure 7).

### Prediction of potential targets of differentially expressed miRNAs

**Figure 8:**
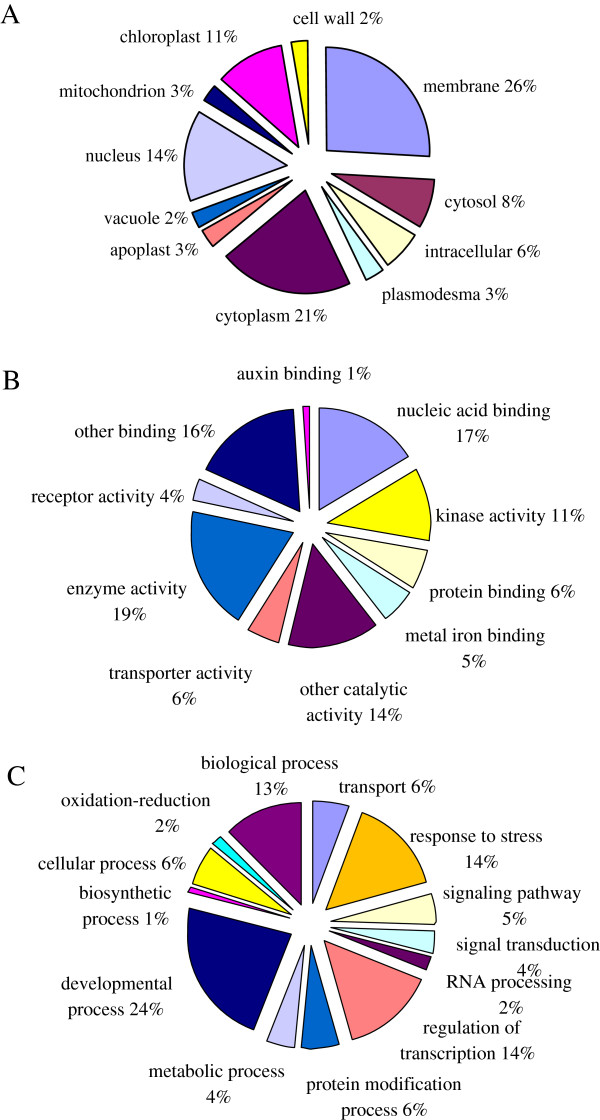
**Gene ontology of the predicted targets for 13 differentially expressed miRNAs.** Categorization of miRNA-target genes was performed according to the cellular component (**A**), molecular function (**B**) and biological process (**C**).

An indication of the genes responsible for pistil abortion was sought by the inspection of 212 potential targets of the 13 differentially expressed miRNAs (Additional file 3). There are three ontologies in Gene Ontology (GO): molecular functions, cellular components and biological processes. GO categories were assigned to all 212 putative targets, according to their cellular components, molecular functions and biological processes (Figure 8). On the basis of molecular function, the genes were finally classified into 10 categories, as shown in Figure 8B. The top three over-represented GO terms are enzyme activity, nucleic acid binding and other binding. Thirteen biological processes were identified, with the three most frequent being developmental process, regulation of transcription and response to stress (Figure 8C).

The high frequency of ‘regulation of transcription’ and ‘response to stress’ terms are easily explainable, since miRNAs are involved in diverse regulatory events [43]. Moreover, many flower developmental genes were also predicted targets of miRNAs. One gene involved in ovule development was further analysed (miR319, miR319a and miR319e and putative target auxin response factor 2 (ARF2) [Ppa022314m]). The results implied the possible roles of miRNAs in the regulation of biological processes involved in pistil abortion.

## Discussion

### High-throughput sequencing of Japanese apricot small RNAs

Most conserved miRNAs in plants have been identified by traditional Sanger sequencing or computational approaches [43-47]. However, a large number of non-conserved or species-specific miRNAs in plants usually accumulate at a lower level than conserved miRNAs, and are typically not easily revealed using traditional sequencing methods [48]. Therefore, the construction and high-throughput sequencing of small RNA libraries seems to be the most efficient method for miRNA identification [36,49]. The aim of this work was to identify the evolutionary known and potentially novel Japanese apricot-specific miRNAs recovered from perfect and imperfect flower bud libraries, and to analyse the differentially expressed miRNAs associated with pistil development. In our study, small RNA libraries of Japanese apricot from the Solexa platform, one of the newly-developed high-throughput sequencing technologies, generated shorter reads (up to 35 bp) but yielded 1–3 million reads per sample. This method has been most popularly employed to discover miRNAs in various organisms. As expected, a large number of reads were generated by our small RNA libraries, of which 61 are known to belong to 24 families. In addition, 61 potentially novel miRNAs/new members of known miRNA families were identified in Japanese apricot.

Taking a broader view of the high-throughput sequencing of small RNAs in Japanese apricot, it was observed that small RNAs of 24 nt dominated the library of unique species, as has been reported for many other plant species, such as *Arabidopsis thaliana*[28], *Citrus trifoliata*[36], *Medicago truncatula*[37,38], *Citrus sativus*[50] and *Citrus Sinensis*[51]. However, the most abundant small RNAs in the imperfect library were 21 nt long. Normally, the length of small RNAs is between 18 nt and 30 nt. Length distribution analysis is a helpful way to assess the composition of small RNA samples. For example, miRNA is normally 21 nt or 22 nt long, whereas siRNA is 24 nt long [52]. The data obtained here imply that the most abundant small RNAs are miRNAs and siRNAs in perfect and imperfect flower buds, respectively.

The overall distribution pattern of small RNAs (21 nt sRNAs = 30.33%, and 24 nt sRNAs = 35.63%) in Japanese apricot is significantly different from that in *Pinus contorta*, a conifer species in which 21 nt RNAs are more abundant (>50%) and 24 nt RNAs are less frequent (2.5%). A striking difference also exists when comparing Japanese apricot small RNAs with monocot species of rice. When compared with eudicotyledon species of sweet orange, the difference is not as noticeable, but still exists, such that 24 nt sRNAs are most frequent (>50%) while 21 nt sRNAs are less common (<20%). These analyses indicate that the small RNA transcriptome is complicated across plant species and can be significantly different between phylogenetically distant plant families.

### miRNAs identified in plant flowers

Arabidopsis miRNAs [27] regulate multiple developmental events. In the past, miRNA identification in flowers using sequencing approaches has been common, and has been applied in the study of *Arabidopsis*[53], tomato [54], orchid [24], *Boechera*[12], maize [55], cotton [49], safflower and grape [56,57]. In this study, high-throughput sequencing of extracts from Japanese apricot flower buds led to the identification of 61 known miRNAs, belonging to 24 families, and 61 potentially novel miRNAs/new members of known miRNA families. Analysis of these revealed that some miRNA families are expressed in the flowers of other plant species, but they are not flower-specific. miR156/miR157, miR166 and miR167 were represented most frequently in the libraries in this study. The involvement of three miRNA families (miR172, miR159/miR319 and miR156) in flowering-time regulation has been recently demonstrated in other investigations [22]. miR164, miR319, miR159 and miR167 specify particular cell types during the later stages of flower development [7]. Furthermore, the comparison of these species’ miRNAs showed that miR156/miR157 and miR172 may be components of a regulatory pathway mediating the transition between the vegetative and reproductive phases in plants. In addition, miR172 regulates stem cell fate and defines the inner boundary of APETALA3 and pistillata expression domains in *Arabidopsis* floral meristems [58]. By targeting APETALA2 and type III homeodomain-leucine zipper (HD-Zip) genes, miR166 regulates the temporal program of floral stem cells [59]. It is believed that miR167, like miR160, targets mRNAs coding for ARF, which are DNA-binding proteins that are thought to control transcription in response to the phytohormone auxin [27,60]. Transcriptional regulation is important for many of the diverse developmental responses to auxin signals, which include cell elongation, division and differentiation in both roots and shoots [61].

### miRNAs possibly involved in the regulation of pistil abortion in imperfect Japanese apricot flower buds

The characterisation and comparative profiling of entire sets of small RNAs (small RNA transcriptome), especially miRNAs, provides the foundation for unraveling the complex miRNA-mediated regulatory networks controlling pistil abortion in imperfect Japanese apricot flower buds. In this study, a number of miRNAs were shown to be differentially expressed between perfect and imperfect flower buds. Compared with the perfect library, four known miRNA genes and three potentially novel miRNA/new members of known miRNA families genes were expressed exclusively in imperfect flower buds. On the other hand, it was found that two potentially novel miRNAs/new members of known miRNA families were perfect-specific. Moreover, a total of seven known miRNAs and six potentially novel miRNAs exhibited significant expression changes between the perfect and imperfect libraries.

The relationship between the sRNA genes and the miRNA target genes is one of the hot spots in the phenomenon of ‘miRNA-associated transitivity’. Target prediction of these differential miRNAs could provide information on the biological processes regulated by miRNA. The annotations of these potential miRNA target genes provided an alternate view of gene regulation of the pistil abortion trait formation in imperfect flower buds. It was discovered that these groups of predicted miRNA-target genes are possibly involved in the pistil abortion trait formation.

The majority of genes encoding transcription factors or F-box proteins have a significant role in the plant development [44,62]. In the present study, it was found that the predicted targets of miR319, miR319a, miR319e, miR160, miR393, miR394, miR6274, miR6295 and miR171d were either transcription factors or F-box proteins. In addition, it is predicted that miR319/miR319a/miR319e target *ARF2* genes and that miR160a targets *ARF16/17*. Auxin regulates a variety of physiological and developmental processes in plants. ARF has been reported to regulate flower and leaf development [8,63,64]. ARF2 is a transcriptional suppressor that has been found to be involved in ethylene, auxin, ABA and brassinosteroid pathways, in order to control the onset of leaf senescence, floral organ abscission and ovule development [64]. ARF2 promotes transitions between multiple stages of *Arabidopsis* development and positively regulates flower development [65]. In this study, the expression of miR319/miR319a/miR319e was shown to be higher in imperfect than in perfect flower buds. Consequently, the expression of ARF2 was repressed by these miRNAs and thus regulated pistil development. Moreover, TCP2 (*TEOSINTE BRANCHED/CYCLOIDEA/PCF*) transcription factor genes and MYB33, which belong to a GAMYB-like family of transcription factors, are also targets of miR319/miR319a/miR319e in our prediction, which agrees with previous reports [10,66]. Therefore, it is conceivable that the over-expression of miR319/miR319a/miR319e may contribute to an increase in imperfect flower ratios in pistil development.

## Conclusions

The present study first comparatively constructed the miRNAomes between perfect and imperfect Japanese apricot flowers and identified 61 known miRNAs belonging to 24 families. Comparative profiling revealed that seven known miRNAs exhibited significant expression differences between perfect and imperfect flower buds. In addition, 61 potentially novel miRNAs/new members of known miRNA families were also identified, by the presence of mature miRNAs and corresponding miRNA*s in the sRNA libraries. Comparative analysis showed that six potentially novel miRNAs were differentially expressed between perfect and imperfect flower buds. Target predictions of the 13 differential miRNAs resulted in 212 target genes. GO annotation revealed that highly-ranked miRNA target genes were those implicated in the developmental process, regulation of transcription and response to stress. These findings, both computational and experimental, provide valuable information for further functional characterisation of miRNAs associated with pistil development in plants.

## Methods

### Plant materials

The frequency of imperfect flowers in Japanese apricot cultivar ‘Daqiandi’ is about 76%. While the pistils of perfect flowers continued to develop, the pistils of imperfect flowers stopped developing in early December and ultimately disintegrated. The present study used these two types of flower buds from this period, from ‘Daqiandi’ trees grown in the ‘National Field Genebank for Japanese apricot’ (Nanjing, Jiangsu Province, China). All the samples were collected and immediately frozen in liquid nitrogen and stored at −80°C.

### Small RNA library construction and high-throughput sequencing

In order to construct small RNA libraries, small RNAs were extracted from perfect and imperfect flower buds using a method based on polyethylene glycol (PEG) precipitation combining CTAB buffer [67]. Two small RNA samples were sequenced by the Beijing Genomics Institute (BGI) (Shenzhen, Guangdong Province, China) using the high-throughput pyrosequencing technology developed by Solexa.

### Bioinformatics analysis of sequencing data

The raw sequences were processed as described by Sunkar *et al.*[25]. Following removal of the vector sequences, modified sequences from 18 nt to 30 nt were used for further analyses. To begin with, rRNA, tRNA, snRNA, snoRNA and material containing the poly-A tail were removed from the sRNA sequences. The remaining sequences were compared with rice and *Arabidopsis* ncRNAs deposited in the NCBI GenBank and Rfam10.0 databases. Then, the unique sRNA sequences were used in a BLASTN search of the miRNA database. Only perfectly matched sequences were considered to be conserved miRNAs.

Potentially novel sequences were identified through alignment with the peach genome sequence on the GDR (http://www.rosaceae.org/species/prunus/peach). Candidate pre-miRNAs were identified by folding the flanking genome sequence of distinct miRNAs using MIREAP [55], followed by a prediction of secondary structure using mFold v3.5 [68]. The criteria chosen for stem-loop hairpins were those described by Meyers *et al.* and Wang *et al.*[42,55].

### Differential expression analyses of miRNAs related to pistil development

The frequency of miRNAs in the two libraries was normalised to one million by the total number of miRNAs in each sample (normalised expression = actual miRNA count/total count of clean reads*1,000,000). Following normalisation, if the miRNA gene expression of two samples was zero, then it was revised to 0.01; if the miRNA gene expression of two samples was less than 1, owing to their too low expression and did not participate in analysis of differential expression.

The fold-change between treatment and control was calculated as: fold-change = log_2_ (imperfect/perfect). The *p-value* was calculated using the formula below [69,70], where: N1 and N2 represent the total counts of clean reads of a given miRNA in the sRNA libraries of perfect and imperfect flower bud samples, respectively; and x and y represent the normalised expression levels of a given miRNA in the sRNA libraries of perfect and imperfect flower bud samples, respectively.

(1) $$p\left(x|y\right)={\left(\frac{N_{2}}{N_{1}}\right)}^{y}\frac{\left(x+\gamma \right)!}{x!\gamma !{\left(1+\frac{N_{2}}{N_{1}}\right)}^{\left(x+\gamma +1\right)}}C\left(\gamma \leq \gamma_{\min}|\text{x}\right)=\sum_{\gamma =0}^{\gamma \leq \gamma_{\min}}p\left(\gamma |\text{x}\right)D\left(\gamma \geq_{\max}|\text{x}\right)=\sum_{\gamma \geq \gamma_{\max}}^{\infty }p\left(\gamma |\text{x}\right)$$

### Prediction of potential target mRNAs for Japanese apricot miRNAs

The rules used for target prediction were based on those suggested by Allen *et al.*[71] and Schwab *et al.*[72]. Putative Japanese apricot miRNAs were first blasted against the peach unigene database on the GDR (http://www.rosaceae.org/species/prunus/peach). BLASTN hits possessing less than four mismatches were chosen as candidate targets. BLASTX was then used to obtain their putative functions.

### Validation of miRNAs by poly(A)-tailed qRT-PCR

The poly(A)-tailed qRT-PCR was carried out as previously reported, with a minor modifications [67,73]. This could not only detect the existence of Japanese apricot miRNAs, but also their expression trends in various organs and tissues. Small RNAs were extracted from Japanese apricot flower buds as described above and used in a 50-μl reaction system for adding poly(A) tails with poly(A) polymerase (NEB, USA). Reverse transcription was performed using poly(A)-tailed small RNAs from Japanese apricot flower buds, such that 1 μg of small RNA was reverse transcribed to cDNA using MLV reverse transcriptase (Promega, Madison, USA) and looped antisense primers [5′-CCAGTAGCGTATGATGAGCACAGAGTCTGAGATCACTCGTAGCGAGG-d(T)_33_-V(A/C/G)N(A/C/G/T)-3′]. According to the manufacturer’s instructions, the mix was incubated at 37°C for 40 minutes. qPCR was performed using SYBR® Green Realtime PCR Master Mix (Toyobo, Osaka, Japan). A list of all the primers used is provided in Additional file 4. For each reaction, 1 μL of diluted cDNA (equivalent to 100 pg of total RNA) was mixed with 10 μL of 2X SYBR Green Reaction Mix (SYBR® Green qRT-PCR Master Mix, Takara). A final volume of 20 μL was achieved by the addition of 5 pmol of the forward and the reverse primers. The conditions for the PCR amplification were as follows: polymerase activation at 95°C for 3 minutes, followed by 40 cycles of 95°C for 20 seconds, 60°C for 20 seconds and 72°C for 45 seconds. The fluorescence signal was measured once every 1°C. Negative PCR controls (no cDNA template) were prepared, in order to detect possible contamination. The specificity of the primer amplicons was tested by the analysis of a melting curve. The CT values were converted into relative copy numbers using a standard curve [44]. The 5 S rRNA was used as a reference gene in the qPCR detection of miRNAs in *Arabidopsis*[56,74]. The data was analysed with an R^2^ >0.998 using the LinRegPCR program [75].

## Competing interests

The authors declare that they have no competing interest.

## Authors’ contributions

TS, XL and WZ were responsible for generating the sRNA data and interpreting the results. TS and XL carried out the qRT-PCR experiments. TS drafted the manuscript. ZZ, LW and ZG participated in the research design and statistical analyses. ZG proposed and supervised the research. All authors read and approved the final manuscript.

## Supplementary Material

Additional file 1 **The predicted hairpin structures of all the potentially novel miRNAs/new members of known miRNA families’ precursors.** The red lightened sequences are mature miRNAs. Click here for file

Additional file 2 Alignments of all reads from each hairpin of potentially novel miRNAs/new members of known miRNA families. Click here for file

Additional file 3 The potential targets of differentially expressed miRNAs. Click here for file

Additional file 4 The primers designed for qRT-PCR. Click here for file
