# Supplementary material for: High-throughput sequencing of small RNAs and analysis of differentially expressed microRNAs associated with pistil development in Japanese apricot
Source: BMC Genomics. 2012 Aug 3;13:371. doi: 10.1186/1471-2164-13-371 (PMC3464595; doi:10.1186/1471-2164-13-371)

Additional file 1: The predicted hairpin structures of all the potentially novel miRNAs/new members of known miRNA families' precursors.

pmu-miR6257

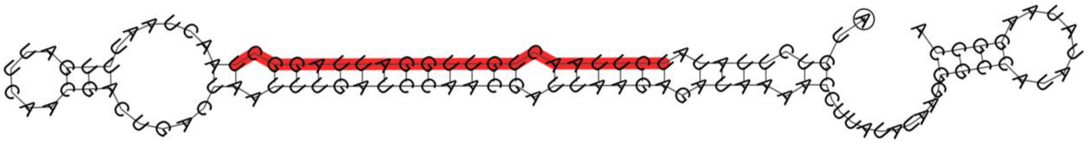

pmu-miR6258

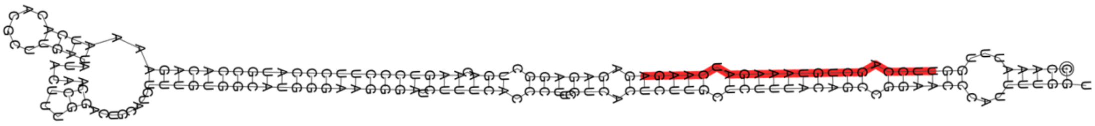

pmu-miR6259

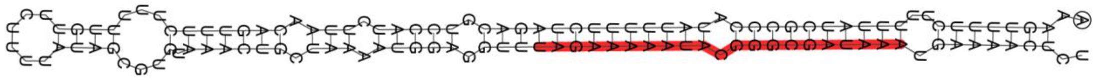

pmu-miR6260

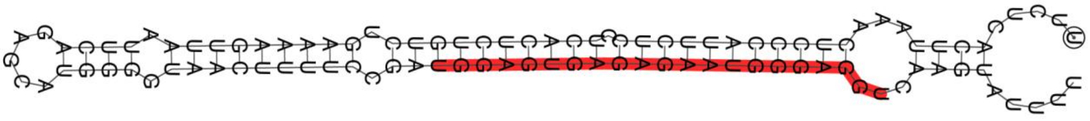

pmu-miR6261

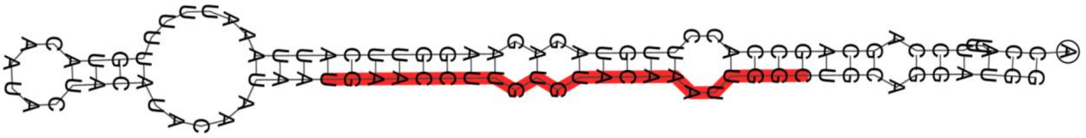

pmu-miR6262

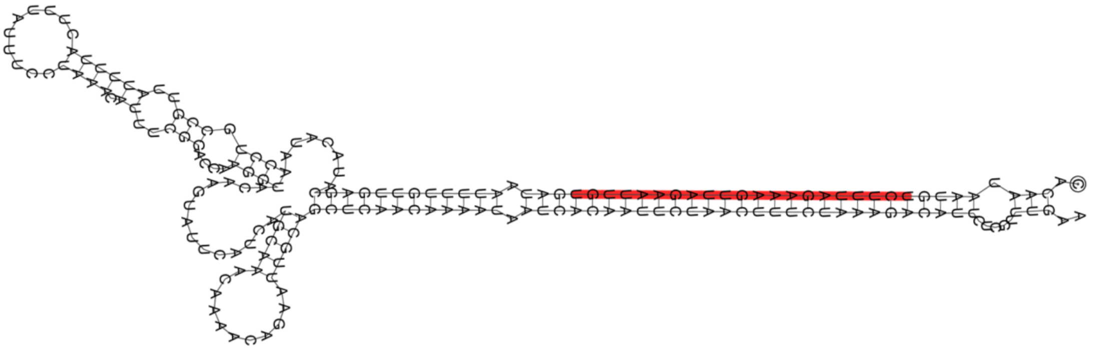

pmu-miR6263

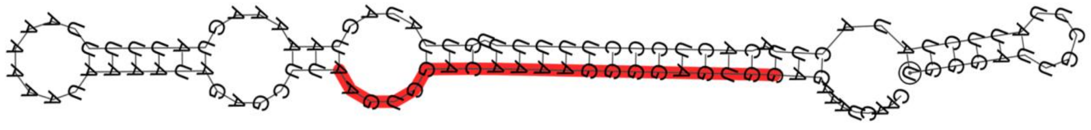

pmu-miR6264

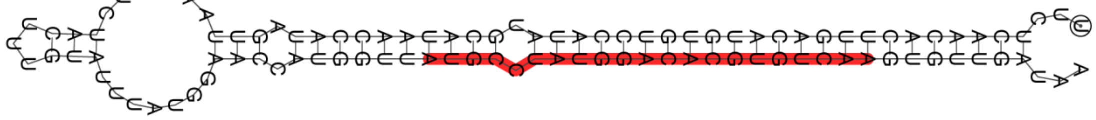

pmu-miR6265

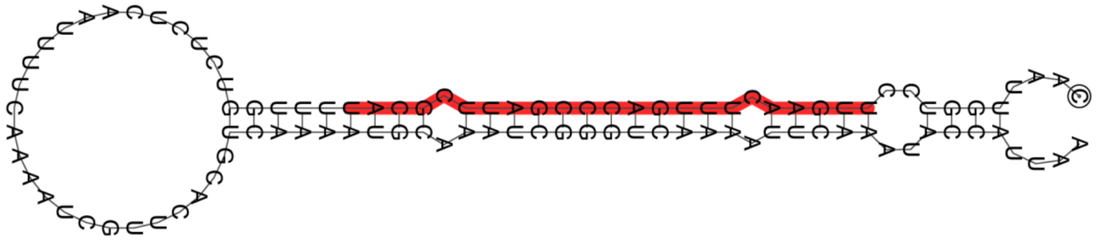

pmu-miR6266a

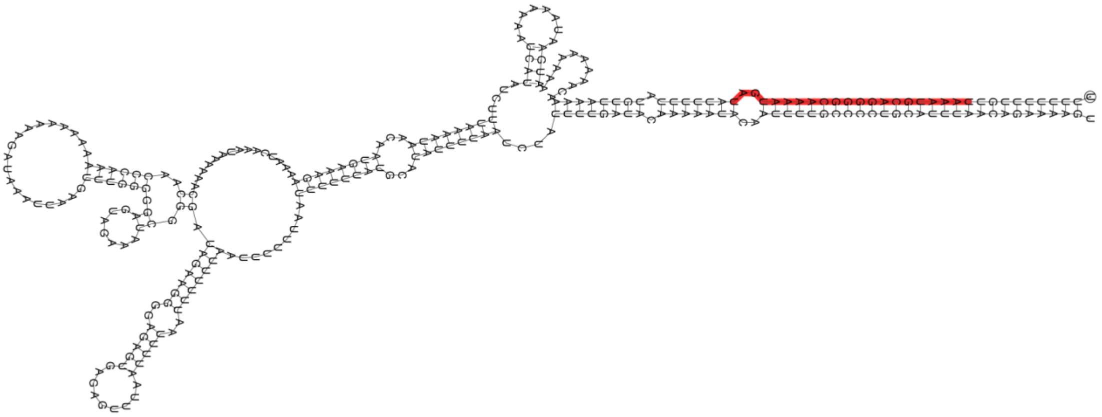

pmu-miR6266b

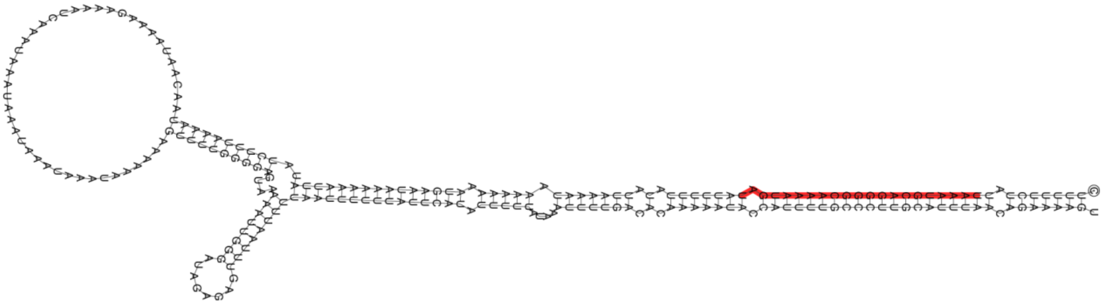

pmu-miR6266c

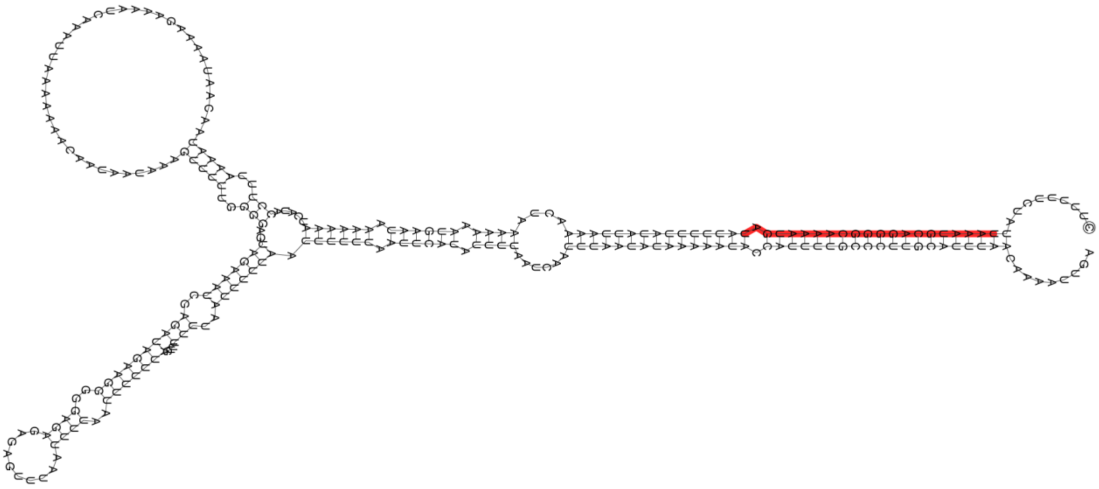

pmu-miR6267a

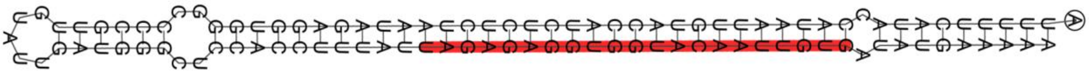

pmu-miR6267b

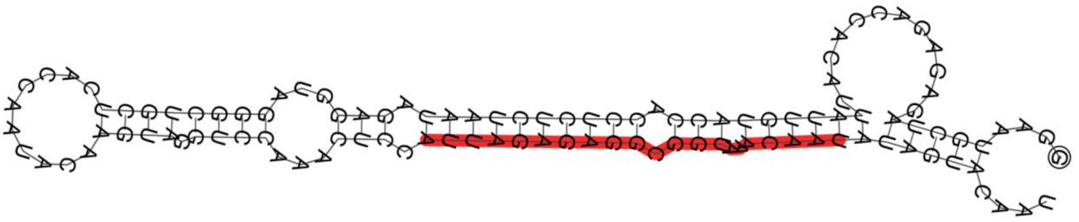

pmu-miR6268

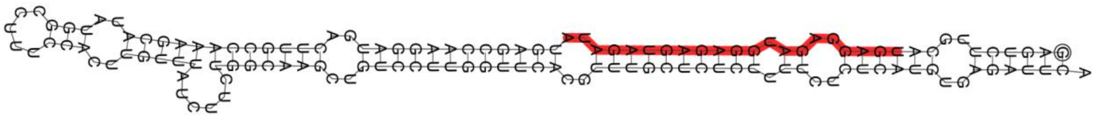

pmu-miR6269

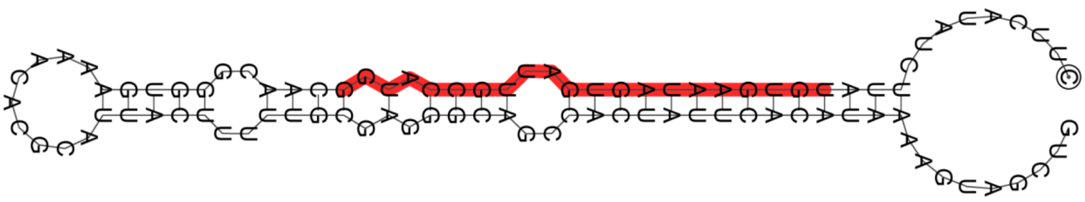

pmu-miR6270

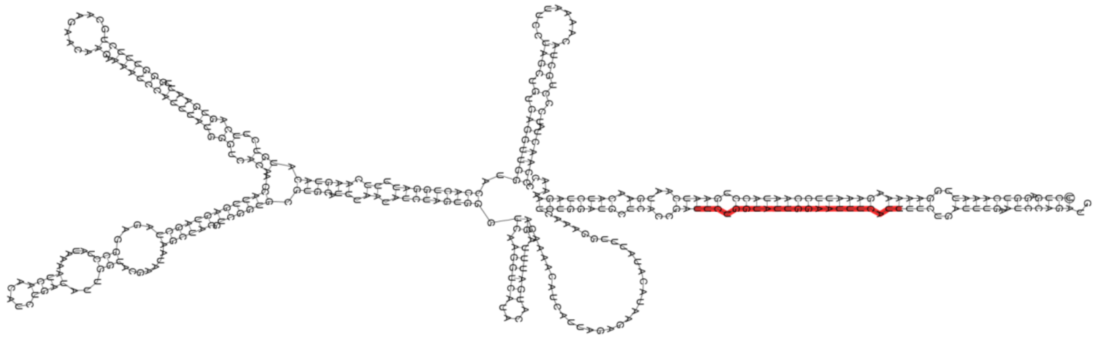

pmu-miR6271

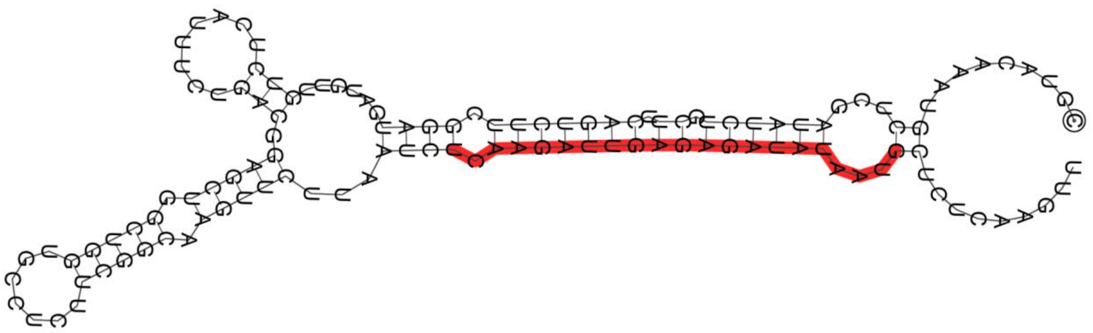

pmu-miR6272

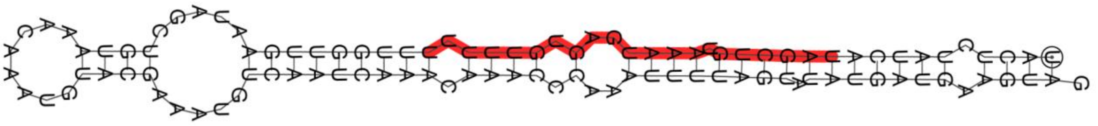

pmu-miR6273

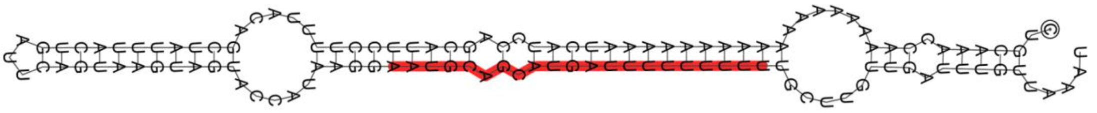

pmu-miR6274

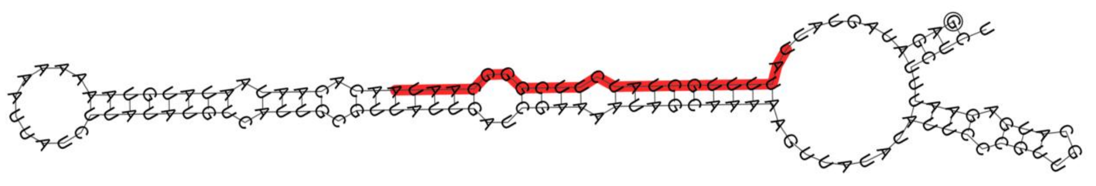

pmu-miR6275

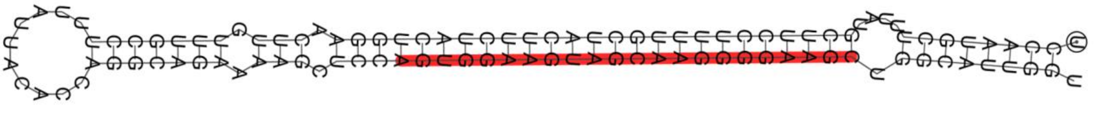

pmu-miR6276

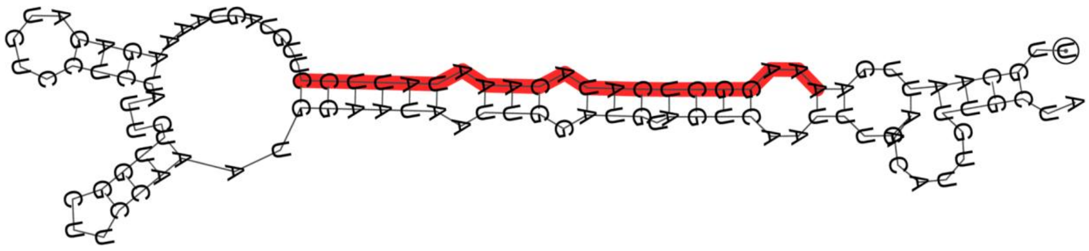

pmu-miR6277

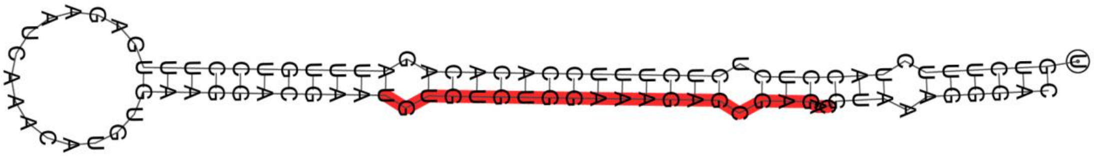

pmu-miR6278

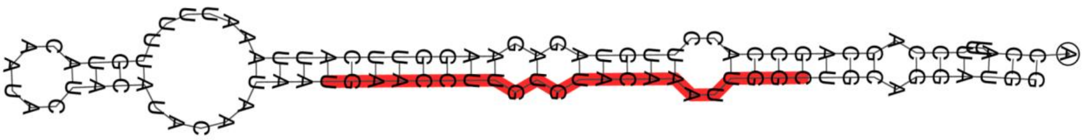

pmu-miR6279

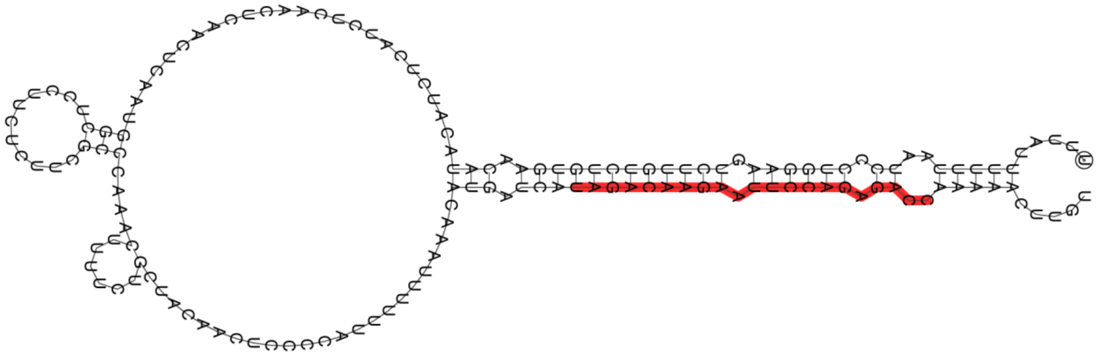

pmu-miR6280

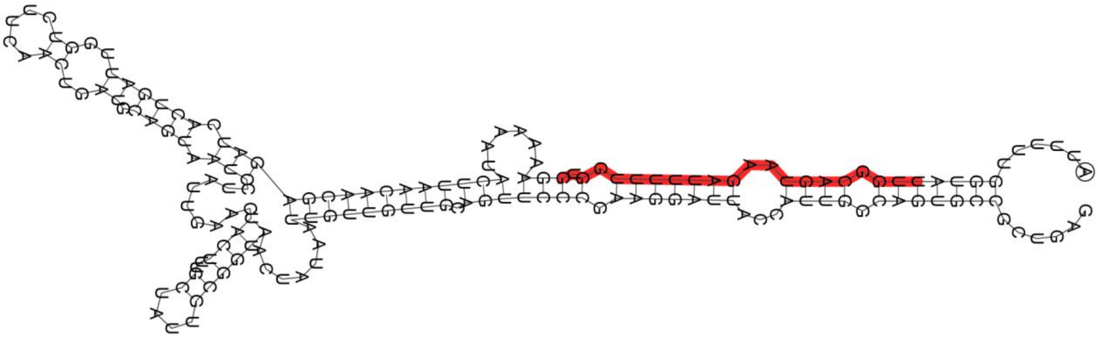

pmu-miR6281

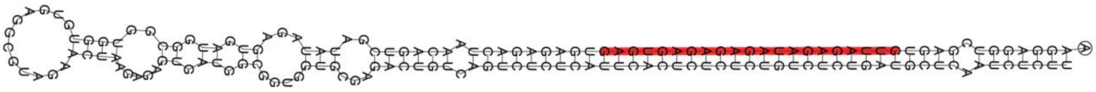

pmu-miR6282

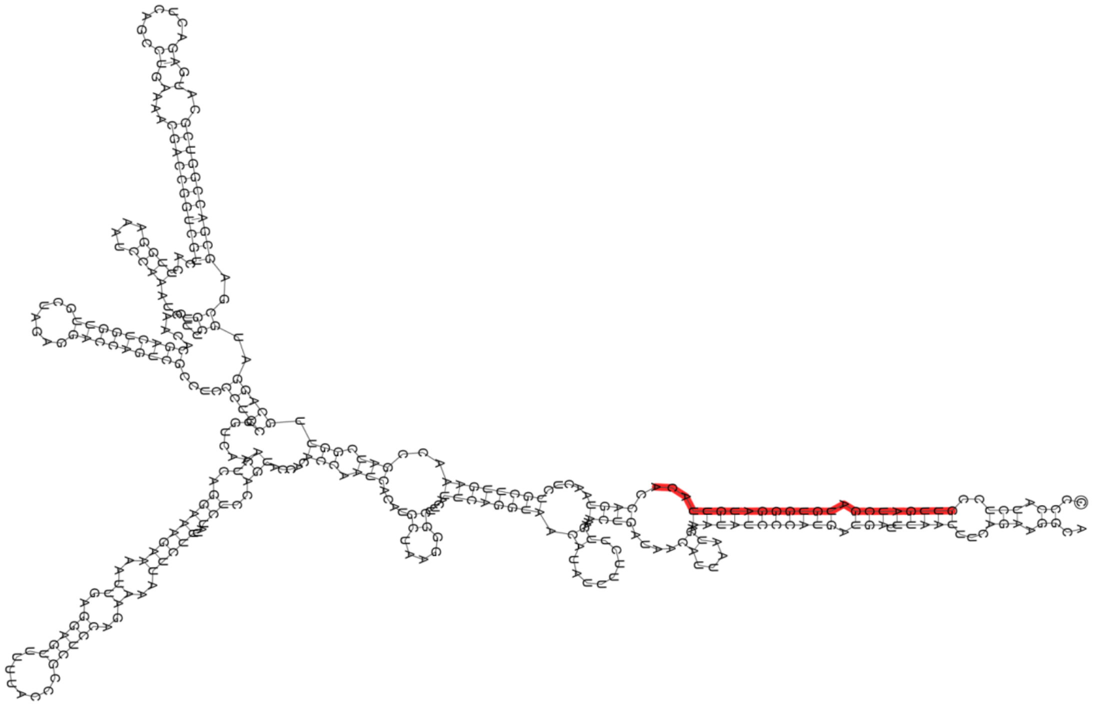

pmu-miR6283

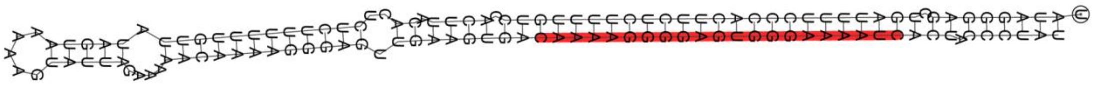

pmu-miR6284

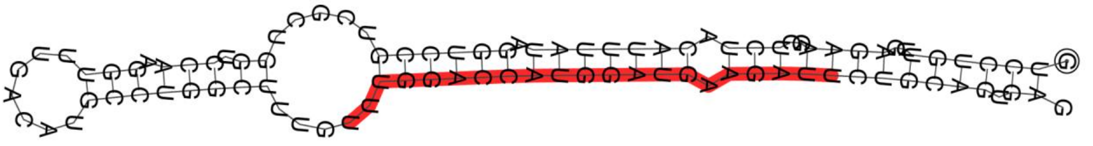

pmu-miR6285

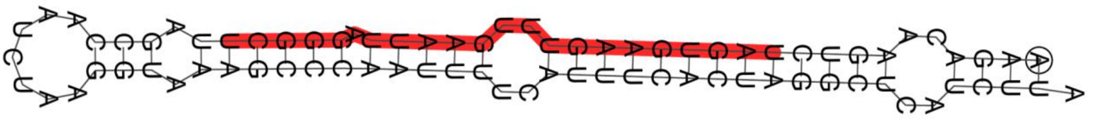

pmu-miR6286

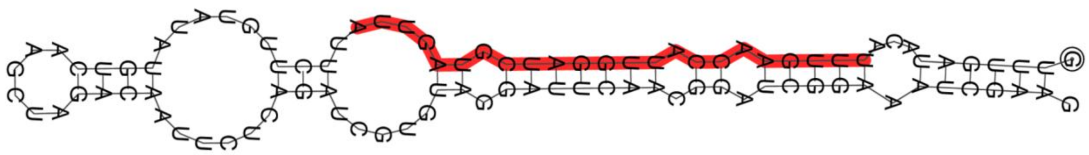

pmu-miR6287

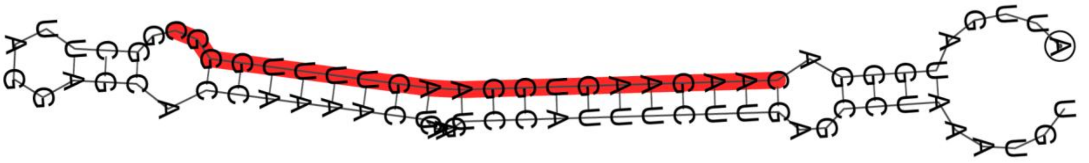

pmu-miR6288

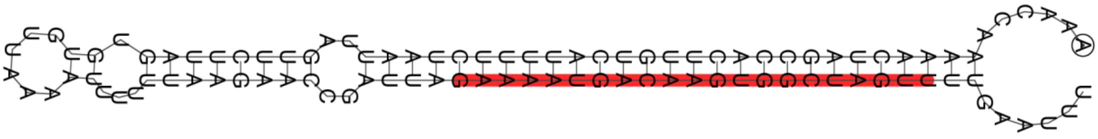

pmu-miR6289

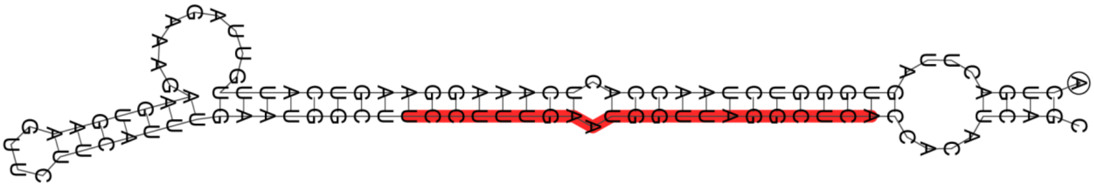

pmu-miR6290

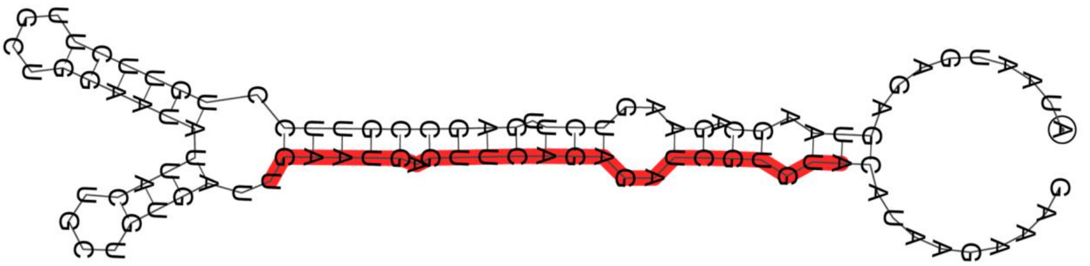

pmu-miR6291a

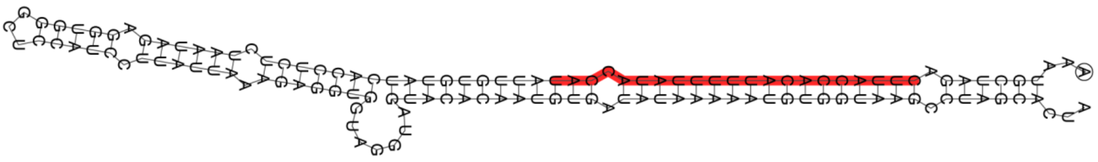

pmu-miR6291b

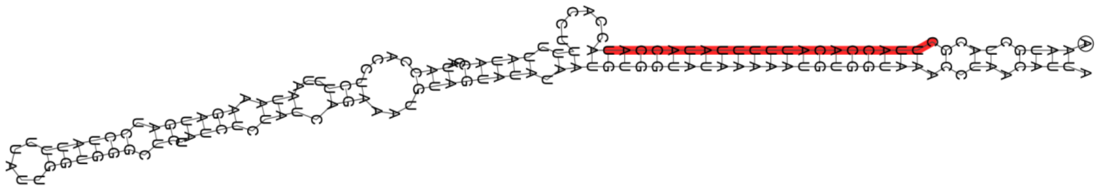

pmu-miR6292

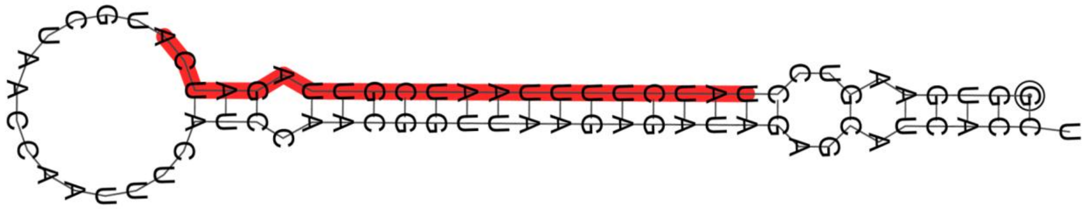

pmu-miR6293

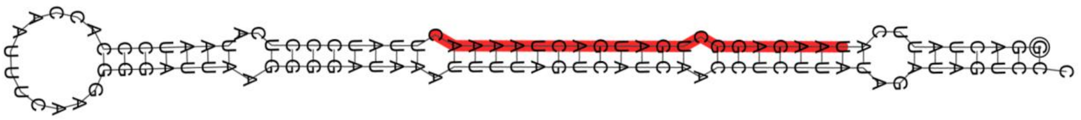

pmu-miR6294

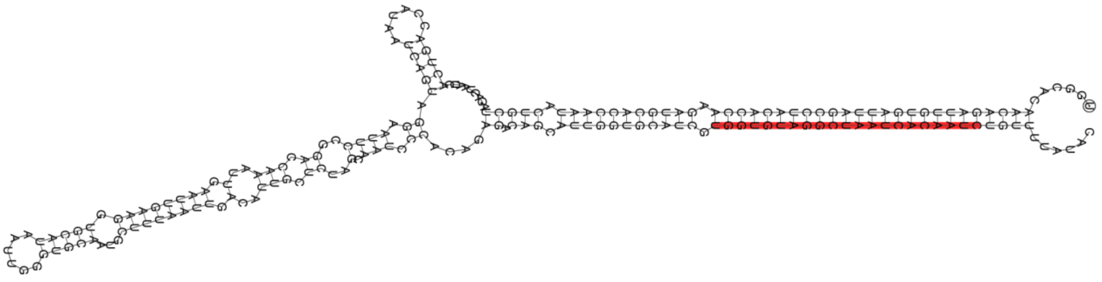

pmu-miR6295

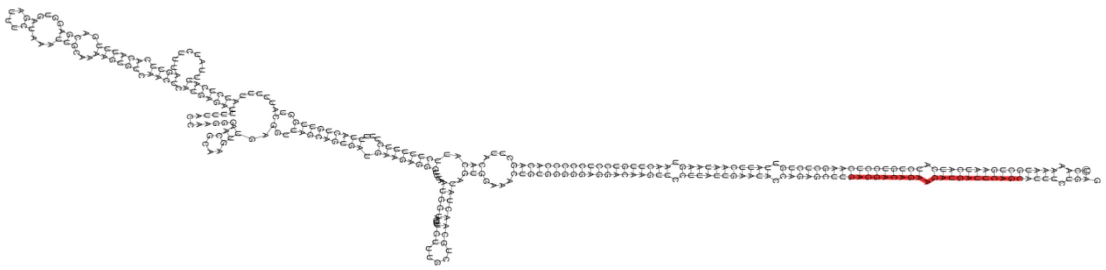

pmu-miR6296

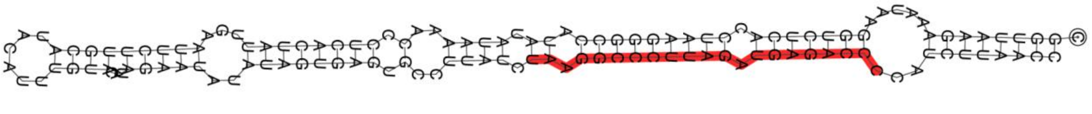

pmu-miR6297a

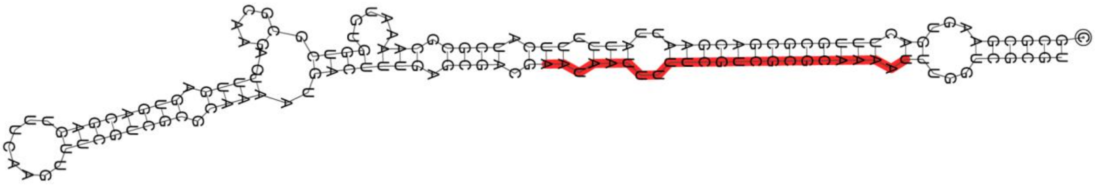

pmu-miR6297b

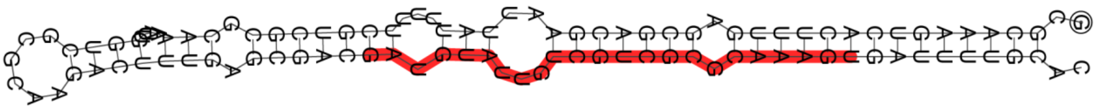

pmu-miR171a

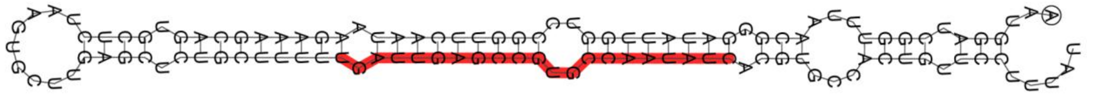

pmu-miR171c

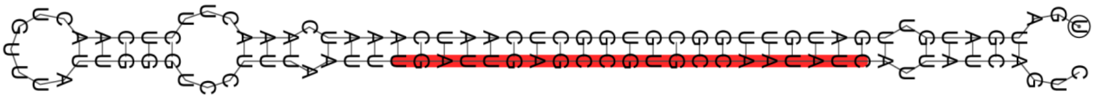

pmu-miR171e

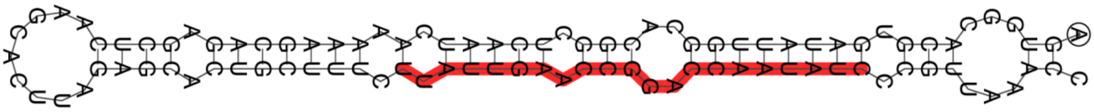

pmu-miR171g

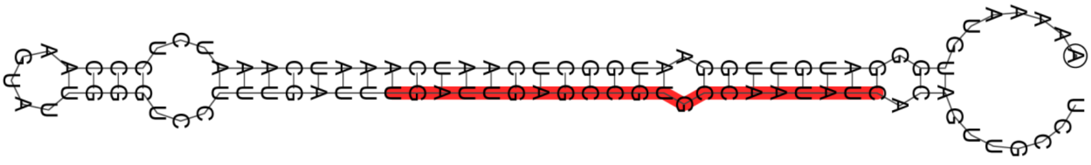

pmu-miR171h

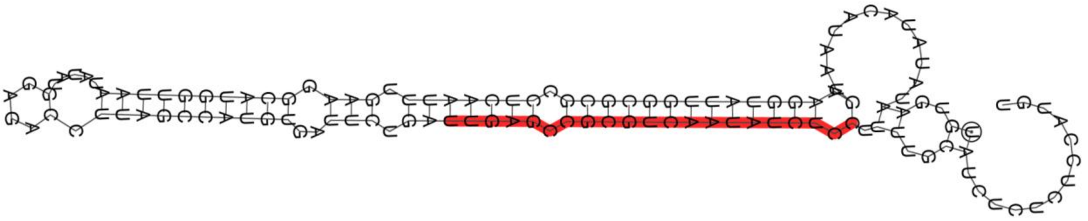

pmu-miR319b

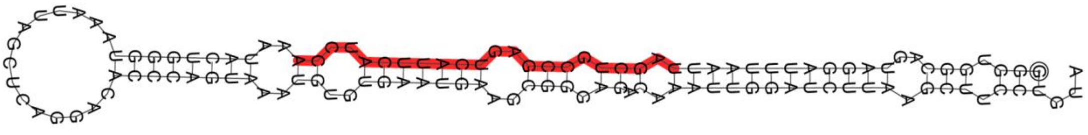

pmu-miR394

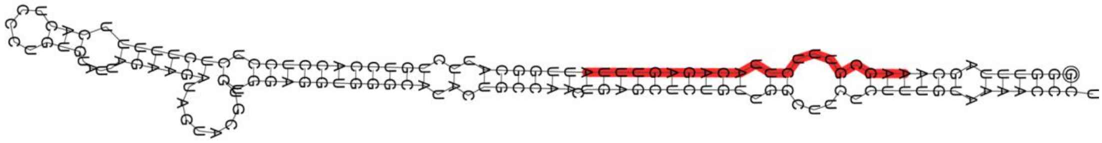

pmu-miR477a

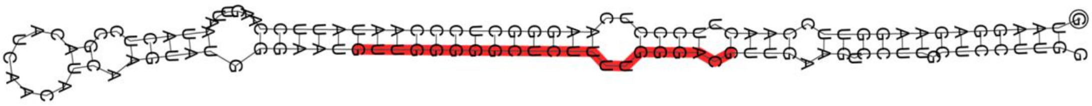

pmu-miR477b

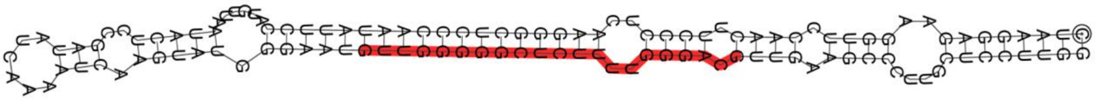

pmu-miR482a

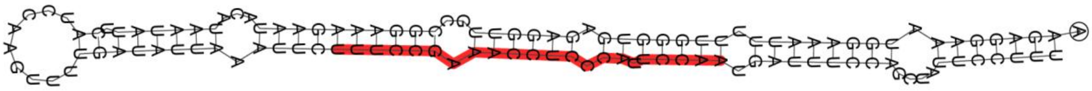

pmu-miR482b

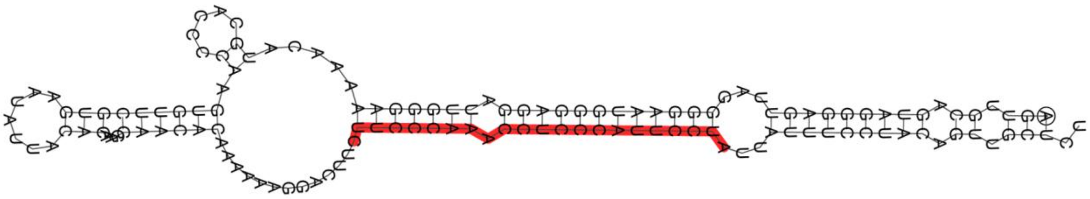

pmu-miR482c

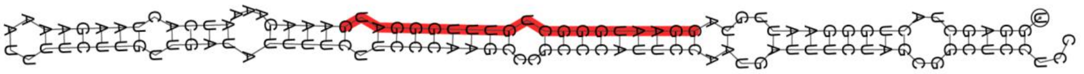

pmu-miR482d

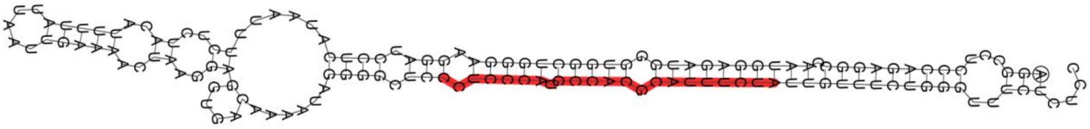

pmu-miR482e

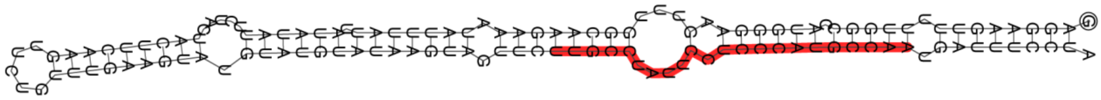

pmu-miR828

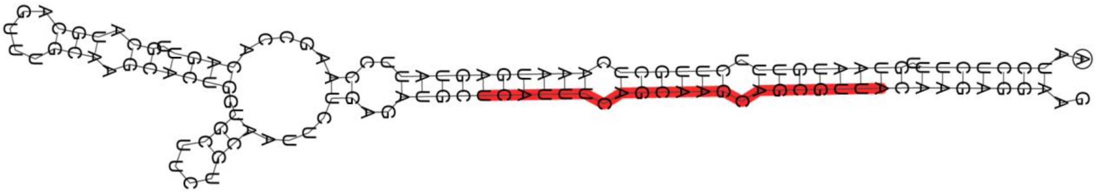

Supplement: Additional file 1 — The predicted hairpin structures of all the potentially novel miRNAs/new members of known miRNA families’ precursors. The red lightened sequences are mature miRNAs. [file 1471-2164-13-371-S1.pdf]
