# Supplementary material for: High-throughput sequencing of small RNAs and analysis of differentially expressed microRNAs associated with pistil development in Japanese apricot
Source: BMC Genomics. 2012 Aug 3;13:371. doi: 10.1186/1471-2164-13-371 (PMC3464595; doi:10.1186/1471-2164-13-371)
Supplement: Additional file 3 — The potential targets of differentially expressed miRNAs. [file 1471-2164-13-371-S3.pdf]

Additional file 2: The potential targets of differentially expressed miRNAs.

miRNA	Target	ID	Target protein
miR319	Ppa000739m	AT5G65700.1	BAM1 Leucine-rich receptor-like protein kinase family protein
	Ppa004612m	AT4G18390.1	TCP2 TEOSINTE BRANCHED 1, cycloidea and PCF transcription factor 2
	Ppa006964m	AT5G59750.2	DHBP synthase RibB-like alpha/beta domain;GTP cyclohydrolase II
	Ppa026535m	AT1G56130.1	Leucine-rich repeat transmembrane protein kinase
	Ppa022825m	AT3G23550.1	MATE effluxfamily protein
	Ppa022749m	AT1G47960.1	C/VIF1 cell wall / vacuolar inhibitor of fructosidase 1
	Ppa022314m	AT5G62000.1	ARF2 auxin response factor 2
	Ppa019782m	AT1G72300.1	Leucine-rich receptor-like protein kinase family protein
	Ppa025945m	AT1G30670.1	basic helix-loop-helix (bHLH) DNA-binding superfamily protein
	ppa008357m	AT11640.1	Thioredoxin superfamilyprotein
miR319a			
	Ppa003628m	AT5G06100.2	MYB33 myb domain protein 33
	Ppa000739m	AT5G65700.1	BAM1 Leucine-rich receptor-like protein kinase family protein
	Ppa004612m	AT4G18390.1	TCP2 TEOSINTE BRANCHED 1, cycloidea and PCF transcription factor 2
	Ppa006964m	AT5G59750.2	DHBP synthase RibB-like alpha/beta domain;GTP cyclohydrolase II
	Ppa003024m	AT1G13750.1	Purple acid phosphatases superfamily protein
	Ppa026535m	AT1G56130.1	Leucine-rich repeat transmembrane protein kinase
	Ppa022825m	AT3G23550.1	MATE efflux family protein
	Ppa022749m	AT1G47960.1	C/VIF1 cell wall / vacuolar inhibitor of fructosidase 1
	Ppa022314m	AT5G62000.1	ARF2 auxin response factor 2
	Ppa019782m	AT1G72300.1	Leucine-rich receptor-like protein kinase family protein
	Ppa025945m	AT1G30670.1	basic helix-loop-helix (bHLH) DNA-binding superfamily protein
	Ppa008357m	AT5G11640.1	Thioredoxin superfamily protein
	Ppa001211m	AT1G56140.1	Leucine-rich repeat transmembrane protein kinase
	Ppa006201m	AT2G20830.2	transferases;folic acid binding
	Ppa010698m	AT4G14550.1	IAA14 indole-3-acetic acid inducible 14
	Ppa016533m	AT2G26150.1	HSFA2 heat shock transcription factor A2
miR319e			
	Ppa003628m	AT5G06100.2	MYB33 myb domain protein 33
	Ppa000739m	AT5G65700.1	BAM1 Leucine-rich receptor-like protein kinase family protein
	Ppa019782m	AT1G72300.1	Leucine-rich receptor-like protein kinase family protein
	Ppa025945m	AT1G30670.1	basic helix-loop-helix (bHLH) DNA-binding superfamily protein
	Ppa001211m	AT1G56140.1	Leucine-rich repeat transmembrane protein kinase
	Ppa004612m	AT4G18390.1	TCP2 TEOSINTE BRANCHED 1, cycloidea and PCF transcription factor 2
	Ppa006964m	AT5G59750.2	DHBP synthase RibB-like alpha/beta domain;GTP cyclohydrolase II
	Ppa003024m	AT1G13750.1	Purple acid phosphatases superfamily protein
	Ppa026535m	AT1G56130.1	Leucine-rich repeat transmembrane protein kinase
	Ppa022825m	AT3G23550.1	MATE efflux family protein
	Ppa022749m	AT1G47960.1	C/VIF1 cell wall / vacuolar inhibitor of fructosidase 1
	Ppa022314m	AT5G62000.1	ARF2 auxin response factor 2
	Ppa008357m	AT5G11640.1	Thioredoxin superfamily protein

miR160a			
	Ppa002710m	AT4G30080.1	ARF16 auxin response factor 16
	Ppa002082m	AT4G30080.1	ARF16 auxin response factor 16
	Ppa002195m	AT4G30080.1	ARF16 auxin response factor 16
	Ppa003136m	AT1G77850.1	ARF17 auxin response factor 17
	Ppa007178m	AT1G21410.1	SKP2A F-box/RNI-like superfamily protein
	Ppa005269m	AT1G21410.1	SKP2A F-box/RNI-like superfamily protein
	Ppa004593m	AT5G44070.1	CAD1 phytochelatin synthase 1 (PCS1)
	Ppa018577m	AT4G15900.1	PRL1 pleiotropic regulatory locus 1
miR393b			
	Ppa003465m	AT3G26810.1	AFB2 auxin signaling F-box 2
	Ppa003344m	AT3G62980.1	TIR1 F-box/RNI-like superfamily protein
	Ppa021102m	AT5G17680.1	disease resistance protein (TIR-NBS-LRR class), putative
	Ppa005384m	AT3G55580.1	Regulator of chromosome condensation (RCC1) family protein
miR394a			
	Ppa004699m	AT1G27340.1	Galactose oxidase/kelch repeat superfamily protein
	Ppa016229m	AT5G06300.1	Putative lysine decarboxylase family protein
	Ppa004770m	AT3G60730.1	Plant invertase/pectin methylesterase inhibitor superfamily
	Ppa024841m	AT1G13570.1	F-box/RNI-like superfamily protein
	Ppa002487m	AT5G62620.1	Galactosyltransferase family protein
	Ppa001198m	AT1G29370.1	Kinase-related protein of unknown function (DUF1296)
	Ppb016096m	AT3G42860.1	zinc knuckle (CCHC-type) family protein
	Ppa019632m	AT1G59720.1	CRR28 Tetratricopeptide repeat (TPR)-like superfamily protein
miR395a			
	Ppa002425m	AT5G10180.1	SULTR2;1 slufate transporter 2;1
	Ppa000024m	AT1G50030.1	TOR target of rapamycin
	Ppa000023m	AT1G50030.1	TOR target of rapamycin
	Ppa000022m	AT1G50030.1	TOR target of rapamycin
	Ppa000635m	AT5G04895.1	DEA(D/H)-box RNA helicase family protein
	Ppa000831m	AT4G14310.1	Transducin/WD40 repeat-like superfamily protein
	Ppa018703m	AT1G43760.1	DNAse I-like superfamily protein
	Ppa020827m	AT1G09820.1	Pentatricopeptide repeat (PPR-like) superfamily protein
	Ppa025910m	AT3G49142.1	Tetratricopeptide repeat (TPR)-like superfamily protein
	Ppa009749m	AT3G60720.1	PDLP8 plasmodesmata-located protein 8
	Ppa009688m	AT5G37660.2	PDLP7 plasmodesmata-located protein 7
	Ppa023145m	AT4G20740.1	Pentatricopeptide repeat (PPR-like) superfamily protein
	Ppa003128m	AT2G01910.1	ATMAP65-6 Microtubule associated protein (MAP65/ASE1) family protein
	Ppa002688m	AT5G52510.1	SCL8 SCARECROW-like 8
	Ppa007945m	AT5G19980.1	GONST4 golgi nucleotide sugar transporter 4
	Ppa002424m	AT4G37870.1	PCK1 phosphoenolpyruvate carboxykinase 1
	Ppa004317m	AT1G30700.1	FAD-binding Berberine family protein
	Ppa003573m	AT2G31340.1	emb1381 embryo defective 1381
	Ppa015277m	AT5G11470.1	bromo-adjacent homology (BAH) domain-containing protein
	Ppa000042m	AT1G20960.1	emb1507 U5 small nuclear ribonucleoprotein helicase, putative

	Ppa007518m	AT3G51880.2	HMGB1 high mobility group B1
	Ppa007828m	AT5G46590.1	NAC096 NAC domain containing protein 96
miR6268			
	Ppa003492m	AT2G05810.1	ARM repeat superfamily protein
	Ppa003437m	AT1G61560.1	MLO6 Seven transmembrane MLO family protein
	Ppa003107m	AT4G21120.1	AAT1 amino acid transporter 1
	Ppa015352m	AT1G27480.1	alpha/beta-Hydrolases superfamily protein
	Ppa000555m	AT3G17340.1	ARM repeat superfamily protein
	Ppa025010m	AT5G19790.1	RAP2.11 related to AP2 11
	Ppa011380m	AT1G12060.1	BAG5 BCL-2-associated athanogene 5
	Ppa001065m	AT1G52360.1	Coatomer, beta\' subunit
	Ppa001045m	AT1G52360.1	Coatomer, beta\' subunit
	Ppa016852m	AT2G32990.1	GH9B8 glycosyl hydrolase 9B8
	Ppa025296m	AT4G37370.1	CYP81D8 cytochrome P450, family 81, subfamily D, polypeptide 8
	Ppa010255m	AT4G27490.1	3\'-5\'-exoribonuclease family protein
	Ppa025516m	AT2G23270.1	
	Ppa014608m	AT3G51680.1	NAD(P)-binding Rossmann-fold superfamily protein
	Ppa010229m	AT3G09050.1	
	Ppa011471m	AT5G26667.1	PYR6 P-loop containing nucleoside triphosphate hydrolases superfamily
	Ppa006889m	AT3G25290.1	Auxin-responsive family protein
	Ppa009095m	AT5G20620.1	UBQ4 ubiquitin 4
	Ppa009116m	AT5G20620.1	UBQ4 ubiquitin 4
	Ppa008113m	AT5G53870.1	ENODL1 early nodulin-like protein 1
	Ppa022441m	AT2G23060.1	Acyl-CoA N-acyltransferases (NAT) superfamily protein
	Ppa006383m	AT5G05320.1	FAD/NAD(P)-binding oxidoreductase family protein
	Ppa004126m	AT5G18590.1	Galactose oxidase/kelch repeat superfamily protein
	Ppa003987m	AT1G65660.1	SMP1 Pre-mRNA splicing Prp18-interacting factor
	Ppa023564m	AT2G41080.1	Tetratricopeptide repeat (TPR)-like superfamily protein
	Ppa026767m	AT1G63130.1	Tetratricopeptide repeat (TPR)-like superfamily protein
	Ppa015739m	AT4G34500.1	Protein kinase superfamily protein
	Ppa019638m	AT4G28890.1	RING/U-box superfamily protein
	Ppa002106m	AT5G64840.1	GCN5 general control non-repressible 5
	Ppa014665m	AT5G05320.1	FAD/NAD(P)-binding oxidoreductase family protein
	Ppa000399m	AT1G67230.1	LINC1 little nuclei1
	Ppa009679m	AT4G24660.1	HB22 homeobox protein 22
	Ppa024165m	AT1G09620.1	ATP binding; leucine-tRNA ligases; aminoacyl-tRNA ligases; nucleotide binding; ATP binding; aminoacyl-tRNA ligases
	Ppa026613m	AT1G23850.1	
	Ppa003946m	AT5G15300.1	Pentatricopeptide repeat (PPR) superfamily protein
	Ppa026657m	AT3G42170.1	BED zinc finger ;hAT family dimerisation domain
	Ppa000624m	AT5G53430.1	SDG29 SET domain group 29
	Ppa026237m	AT3G03550.1	RING/U-box superfamily protein
	Ppa001740m	AT2G27090.1	Protein of unknown function (DUF630 and DUF632)
	Ppa005469m	AT1G04990.1	Zinc finger C-x8-C-x5-C-x3-H type family protein

	Ppa003967m	AT3G15620.1	UVR3 DNA photolyase family protein
	Ppa023353m	AT3G42170.1	BED zinc finger ;hAT family dimerisation domain
	Ppa023798m	AT1G12700.1	ATP binding;nucleic acid binding;helicases
	Ppa014933m	AT5G15300.1	Pentatricopeptide repeat (PPR) superfamily protein
	Ppa026995m	AT3G20960.1	CYP705A33 cytochrome P450, family 705, subfamily A, polypeptide 33
	Ppa022097m	AT2G17525.1	Pentatricopeptide repeat (PPR) superfamily protein
	Ppa004195m	AT3G49000.1	RNA polymerase III subunit RPC82 family protein
	Ppa021893m	AT5G05320.1	FAD/NAD(P)-binding oxidoreductase family protein
	Ppb012607m		
	Ppa023652m	AT5G45160.1	Root hair defective 3 GTP-binding protein (RHD3)
	Ppa006387m	AT5G05320.1	FAD/NAD(P)-binding oxidoreductase family protein
	Ppa023380m	AT1G32120.1	
	Ppa025705m	AT1G23850.1	
	Ppa008775m	AT1G08315.1	ARM repeat superfamily protein
	Ppa004391m	AT3G26990.1	ENTH/VHS family protein
	Ppa026817m	AT3G22670.1	Pentatricopeptide repeat (PPR) superfamily protein
miR6274			
	Ppa014136m		
	Ppa011211m	AT5G19830.1	Peptidyl-tRNA hydrolase family protein
	Ppa016691m	AT1G71680.1	Transmembrane amino acid transporter family protein
	Ppa005516m	AT2G28130.1	
	Ppa019955m	AT3G07870.1	F-box and associated interaction domains-containing protein
	Ppb024849m	AT5G42905.1	Polynucleotidyl transferase, ribonuclease H-like superfamily protein
	Ppa016131m	AT1G72300.1	Leucine-rich receptor-like protein kinase family protein
	Ppa018920m	AT5G43470.1	RPP8 Disease resistance protein (CC-NBS-LRR class) family
	Ppa005583m	AT5G41800.1	Transmembrane amino acid transporter family protein
	Ppa002159m	AT5G06740.1	Concanavalin A-like lectin protein kinase family protein
	Ppa004234m	AT5G03070.1	IMPA-9 importin alpha isoform 9
	Ppa001639m	AT4G09020.1	ISA3 isoamylase 3
	Ppa021306m	AT1G15030.1	Protein of unknown function (DUF789)
	Ppa000156m	AT2G34660.1	MRP2 multidrug resistance-associated protein 2
	Ppa011557m	AT4G28400.1	Protein phosphatase 2C family protein
	Ppa002399m	AT5G35700.1	FIM2 fimbrin-like protein 2
	Ppa008670m	AT2G29070.2	
miR6295			
	Ppa003799m	AT3G22440.1	FRIGIDA-like protein
	Ppa012230m	AT1G09320.1	agenet domain-containing protein
	Ppa003837m	AT4G19180.1	GDA1/CD39 nucleoside phosphatase family protein
	Ppa023910m	AT4G39370.1	UBP27 ubiquitin-specific protease 27
	Ppa009741m	AT3G58600.1	Adaptin ear-binding coat-associated protein 1 NECAP-1
	Ppa000357m	AT1G18270.2	ketose-bisphosphate aldolase class-II family protein
	Ppa007339m	AT1G28220.1	PUP3 purine permease 3
	Ppa000076m	AT4G13750.1	NOV Histidine kinase-, DNA gyrase B-, and HSP90-like ATPase family protein

	Ppa008693m	AT1G80130.1	Tetratricopeptide repeat (TPR)-like superfamily protein
	Ppa009781m	AT3G20970.1	NFU4 NFU domain protein 4
	Ppa004286m	AT2G01070.1	Lung seven transmembrane receptor family protein
	Ppa006763m	AT5G02850.1	hydroxyproline-rich glycoprotein family protein
	Ppa006205m	AT5G58230.1	MSI1 Transducin/WD40 repeat-like superfamily protein
	Ppa001776m	AT1G15690.1	AVP1 Inorganic H pyrophosphatase family protein
	Ppa021050m	AT1G48570.1	zinc finger (Ran-binding) family protein
	Ppa016261m	AT3G14690.1	CYP72A15 cytochrome P450, family 72, subfamily A, polypeptide 15
	Ppa008101m	AT1G10070.1	BCAT-2 branched-chain amino acid transaminase 2
	Ppa001055m	AT2G47980.1	SCC3 sister-chromatid cohesion protein 3
	Ppa010532m	AT4G19150.1	Ankyrin repeat family protein
	Ppa022131m	AT2G33030.1	RLP25 receptor like protein 25
	Ppa005781m	AT5G54130.2	Calcium-binding endonuclease/exonuclease/phosphatase family
	Ppa004952m	AT5G63080.1	2-oxoglutarate (2OG) and Fe(II)-dependent oxygenase superfamily protein
	Ppa004360m	AT1G51340.2	MATE efflux family protein
	Ppa002221m	AT2G16365.1	F-box family protein
	Ppa000069m	AT5G02310.1	PRT6 proteolysis 6
	Ppa000460m	AT1G20920.1	P-loop containing nucleoside triphosphate hydrolases superfamily protein
	Ppa001581m		
	Ppa002620m	AT4G34110.1	PAB2 poly(A) binding protein 2
	Ppa002618m	AT4G34110.1	PAB2 poly(A) binding protein 2
	Ppa015277m	AT5G11470.1	bromo-adjacent homology (BAH) domain-containing protein
	Ppa022784m	AT5G66520.1	Tetratricopeptide repeat (TPR)-like superfamily protein
	Ppa022971m	AT1G16130.1	WAKL2 wall associated kinase-like 2
	Ppa021700m	AT3G59410.1	GCN2 protein kinase family protein
	Ppa003983m	AT5G58320.2	Kinase interacting (KIP1-like) family protein
	Ppa000031m	AT5G44800.1	CHR4 chromatin remodeling 4
	Ppa020352m	AT1G34340.1	alpha/beta-Hydrolases superfamily protein
	Ppa007163m	AT1G48300.1	
miR171d			
	Ppa001824m	AT4G27290.1	S-locus lectin protein kinase family protein
	Ppa001561m	AT4G00150.1	HAM3 GRAS family transcription factor
	Ppa001781m	AT4G00150.1	HAM3 GRAS family transcription factor
	Ppa000996m	AT1G44900.1	MCM2 minichromosome maintenance (MCM2/3/5) family protein
	Ppa016845m	AT3G47680.1	DNA binding
miR319b			
	Ppa004289m	AT5G40440.1	MKK3 mitogen-activated protein kinase kinase 3
	Ppa000132m	AT3G11130.1	Clathrin, heavy chain
	Ppa026169m	AT5G17680.1	disease resistance protein (TIR-NBS-LRR class), putative
	Ppa012174m	AT5G38430.1	Ribulose bisphosphate carboxylase (small chain) family protein
	Ppa016564m	AT2G44200.1	CBF1-interacting co-repressor CIR, N-terminal;Pre-mRNA splicing factor
miR482c			
	Ppa009570m	AT3G53690.1	RING/U-box superfamily protein
	Ppa005150m	AT2G41540.1	GPDHC1 6-phosphogluconate dehydrogenase family protein

Ppa009473m	AT2G26660.1	SPX2 SPX domain gene 2
Ppa011036m	AT3G60080.1	RING/U-box superfamily protein
Ppa004552m	AT4G21390.1	B120 S-locus lectin protein kinase family protein
Ppa000532m	AT3G54440.1	glycoside hydrolase family 2 protein
Ppa026041m		
Ppa011801m	AT3G03420.1	Ku70-binding family protein
Ppa000751m	AT1G20760.1	Calcium-binding EF hand family protein
Ppa000034m	AT1G36160.1	ACC1 acetyl-CoA carboxylase 1
Ppa000508m	AT3G54440.1	glycoside hydrolase family 2 protein
