# Supplementary material for: High-throughput sequencing of small RNAs and analysis of differentially expressed microRNAs associated with pistil development in Japanese apricot
Source: BMC Genomics. 2012 Aug 3;13:371. doi: 10.1186/1471-2164-13-371 (PMC3464595; doi:10.1186/1471-2164-13-371)
Supplement: Additional file 4 — The primers designed for qRT-PCR. [file 1471-2164-13-371-S4.pdf]

Additional file 3: The primers designed for qRT-PCR.

Primer Code No	Primer Sequence (5'-3')	Size of primers (bp)
miR319a	TTGGACTGAAGGGAGCTCCCT	21
miR160a	TGCCTGGCTCCCTGTATGCCA	21
miR393a	TCCAAAGGGATCGCATTGATCC	22
miR394a	TTGGCATTCTGTCCACCTCC	20
novel 16	GTTCGATGCCACGTCGCC	18
novel 18	TGATTGAGCCGTGCCAATATC	21
novel 24	TGAGGAGATGGAGAGTAGATAAA	23
novel 33	TATTTTGCTATCTTCGGGCAATA	23
novel 35	TAGCTGCCGAGTCATTCATCCA	22
novel 53	TGATTGAGCCGTGCCAATATC	21
novel 63	TAGCTGCCGAGTCATTCATCCA	22
novel 64	GAGGACAGAAGATGATTCAGC	21
reverse	CCAGTAGCGTATGATGAGCA	20
5SrRNAf	CTCGGCAACGGATATCTCGGCTCT	24
5SrRNAr	CTAATGGCTTGGGGCGCAACTTG	23
